# A computational model of postprandial adipose tissue lipid metabolism derived using human arteriovenous stable isotope tracer data

**DOI:** 10.1371/journal.pcbi.1007400

**Published:** 2019-10-03

**Authors:** Shauna D. O’Donovan, Michael Lenz, Roel G. Vink, Nadia J. T. Roumans, Theo M. C. M. de Kok, Edwin C. M. Mariman, Ralf L. M. Peeters, Natal A. W. van Riel, Marleen A. van Baak, Ilja C. W. Arts

**Affiliations:** 1 Maastricht Centre for Systems Biology (MaCSBio), Maastricht University, Maastricht, The Netherlands; 2 Division of Human Health and Nurtrition, Wageningen University, Wageningen, The Netherlands; 3 Institute of Organismic and Molecular Evolution, Johannes Gutenberg University Mainz, Mainz, Germany; 4 Preventive Cardiology and Preventative Medicine - Center for Cardiology, University Medical Center of the Johannes Gutenberg University Mainz, Mainz, Germany; 5 Dept. Human Biology, NUTRIM School of Nutrition and Translational Research in Metabolism, Maastricht University, Maastricht, The Netherlands; 6 Dept. Toxicogenomics, GROW School for Oncology and Developmental Biology, Maastricht University, Maastricht, The Netherlands; 7 Dept. Data Science and Knowledge Engineering, Maastricht University, Maastricht, The Netherlands; 8 Dept. Biomedical Engineering, Eindhoven University of Technology, Eindhoven, The Netherlands; 9 Dept. Epidemiology, CARIM School for Cardiovascular Disease, Maastricht University, Maastricht, The Netherlands; University of Connecticut School of Medicine, UNITED STATES

## Abstract

Given the association of disturbances in non-esterified fatty acid (NEFA) metabolism with the development of Type 2 Diabetes and Non-Alcoholic Fatty Liver Disease, computational models of glucose-insulin dynamics have been extended to account for the interplay with NEFA. In this study, we use arteriovenous measurement across the subcutaneous adipose tissue during a mixed meal challenge test to evaluate the performance and underlying assumptions of three existing models of adipose tissue metabolism and construct a new, refined model of adipose tissue metabolism. Our model introduces new terms, explicitly accounting for the conversion of glucose to glyceraldehye-3-phosphate, the postprandial influx of glycerol into the adipose tissue, and several physiologically relevant delays in insulin signalling in order to better describe the measured adipose tissues fluxes. We then applied our refined model to human adipose tissue flux data collected before and after a diet intervention as part of the Yoyo study, to quantify the effects of caloric restriction on postprandial adipose tissue metabolism. Significant increases were observed in the model parameters describing the rate of uptake and release of both glycerol and NEFA. Additionally, decreases in the model’s delay in insulin signalling parameters indicates there is an improvement in adipose tissue insulin sensitivity following caloric restriction.

## Author summary

The adipose tissue is no longer considered a metabolically quiescent tissue. Disturbances in non-esterified fatty acid (NEFA) metabolism leading to ectopic fat deposition have been associated with the development of insulin resistance. In recent years, the use of stable isotope tracers coupled with arteriovenous sampling across tissue depots has greatly improved our knowledge of postprandial NEFA dynamics. In this study, we make use of arteriovenous measurements collected across the abdominal subcutaneous adipose tissue in humans during a high fat mixed meal to evaluate three existing computational models of adipose tissue metabolism. As the three models included in this study were not capable of fully describing the measured adipose tissue fluxes, we present a new model of human *in vivo* adipose tissue metabolism, introducing novel terms such as the postprandial uptake of glycerol. We then utilised our refined model to quantify the effect of caloric restriction on adipose tissue metabolism by fitting the model to mixed meal challenge test data collected before and after a weight-loss intervention. Parameter estimates indicate an increase in the rates of glycerol and NEFA release coupled with a decrease in the delay of insulin signalling for all reactions, suggesting improved insulin sensitivity following caloric restriction.

## Introduction

The adipose tissue plays a key role in the regulation of plasma triglyceride and non-esterified fatty acid (NEFA) concentrations [1, 2]. In the fasting state, lipolysis of triglycerides stored in the adipose tissue delivers NEFA to the plasma, where it can be taken up by other tissues, including skeletal muscle, heart, and liver [3]. In the postprandial state dietary derived chylomicron-triglycerides are removed from the circulation by insulin stimulated hydrolysis by lipoprotein lipase (LPL) at the endothelial cell wall [4]. NEFA released by LPL lipolysis is primarily taken up by the adipose tissue and may subsequently be stored as triglyceride [5]. Disturbances in the regulation of lipid metabolism by the adipose tissue can lead to raised plasma NEFA concentrations resulting in ectopic fat deposition. The accumulation of fat in insulin sensitive tissues such as skeletal muscle and liver has been related to the development of insulin resistance and, consequently, increases the risk for developing Type 2 Diabetes and cardiovascular disease [6–9]. The contribution of disturbed NEFA metabolism to the development of insulin resistance highlights the need to include adipose tissue NEFA metabolism when investigating disturbances in the glucose-insulin regulatory system during disease development [10].

Several computational models exist which describe adipose tissue NEFA metabolism; from smaller models focussing on NEFA dynamics between the plasma and adipose tissue alone [11, 12] to larger models describing several metabolites across multiple tissues [10, 13, 14]. Typically, these models have been parameterised using values reported in literature and their performance assessed by comparison with measured plasma metabolite concentrations during an oral glucose or mixed meal challenge test. However, with traditional venous sampling it is not possible to differentiate between the contributions of individual tissues (i.e. liver or skeletal muscle) to the whole body plasma metabolite concentration. Consequently, it is not possible to fully assess the structure of the model and assumptions upon which the model has been constructed. Therefore, more detailed measurements are needed, providing information about contributions of individual tissues to postprandial changes in plasma NEFA concentrations.

In recent years, new quantitative knowledge has been gained on human adipose tissue NEFA dynamics in both the fasting and postprandial state through the use of stable isotope tracers coupled with arteriovenous sampling [5, 15, 16]. The simultaneous sampling of blood from an artery (or arterialised hand vein) and a vein draining a specific tissue depot (e.g. the abdominal subcutaneous adipose tissue) allows for the calculation of metabolite fluxes across this tissue. In combination with the use of stable isotope tracers, it is now possible to further untangle postprandial lipid metabolism and to quantify rates of appearance of triglyceride in the plasma, rates of LPL lipolysis of lipoprotein derived triglyceride, and spill-over of LPL derived NEFA into the plasma, while also providing insights into the behaviour of secondary metabolites such as glycerol.

Arteriovenous measurements provide invaluable information in the evaluation and refinement existing models of adipose tissue fatty acid metabolism. One example where such data has been successfully used is the 2008 study of Kim et al. [12], who modelled adipose tissue metabolism with a focus on the regulation of lipolysis. However, their model focused primarily on the fasting state, as well as the effect of an epinephrine infusion, and did not consider the postprandial state nor the influence of insulin.

In the present study, we used human in vivo measurements of net triglyceride, NEFA, glucose, and glycerol fluxes across the abdominal subcutaneous adipose tissue along with arterial insulin collected in the fasting and postprandial state during a high fat mixed meal challenge test to evaluate three existing models of postprandial adipose tissue dynamics. The inclusion of a palmitate stable isotope tracer in the meal provides a first opportunity to evaluate model terms describing the postprandial spill-over of LPL derived NEFA on human data. Ultimately, as no existing model could sufficiently describe the calculated metabolite fluxes, we construct a new refined model of postprandial adipose tissue metabolism which could be parameterised by data; introducing novel terms explicitly accounting for the conversion of glucose to G-3-P for use in re-esterification and the postprandial uptake of glycerol into the adipose tissue. The resulting model was then used to quantitatively estimate the impact of caloric restriction induced weight loss on subcutaneous adipose tissue fatty acid dynamics in a population of sixteen overweight or obese individuals that participated in the Yoyo study [17]. The model also allows for the prediction of the dynamic response of several reactions, which were not directly measured, to the ingestion of a meal (e.g. uptake and release of NEFA and glycerol by the adipose compartment), providing additional insights into the alterations in adipose tissue metabolism following caloric restriction which contribute to the observed changes in the calculated adipose tissue fluxes.

## Materials and methods

### Study design and work-flow

**Fig 1:**
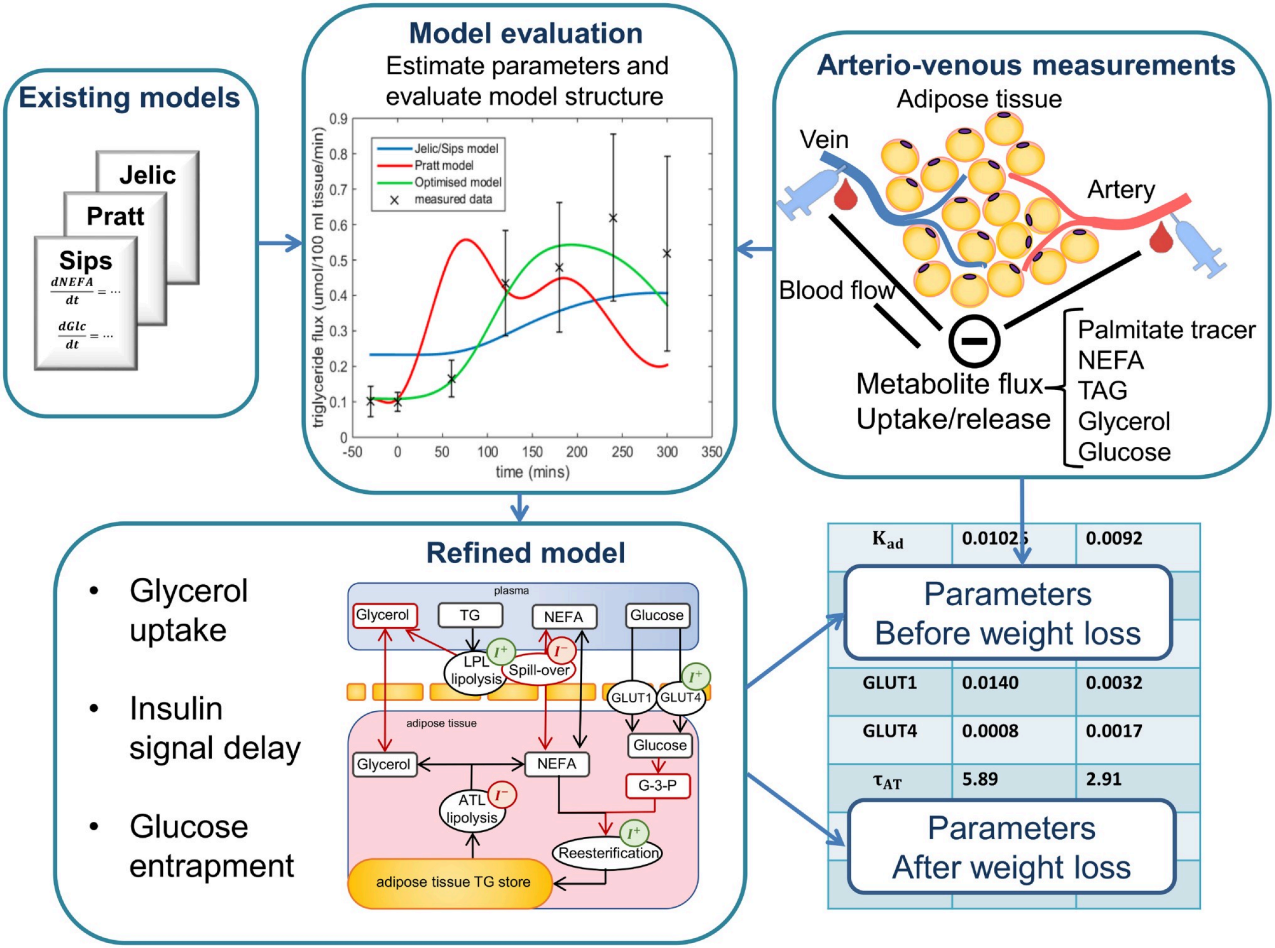
Work-flow for comparison of existing model terms and construction of refined model. Jelic, Pratt, and Sips model terms are compared for each metabolite flux separately using the postprandial arteriovenous tracer measurements from the Yoyo study. Existing model terms are first evaluated based on their ability to describe the experimentally measured data. In addition, as the refined model is constructed with the aim that parameters could be estimated directly from measured data, existing model terms are also compared using identifiability analysis, using the Profile Likelihood, sensitivity analysis, and statistical evaluation using Akaike Information Criterion. The refined model of adipose tissue metabolism is constructed either by selecting the best fitting of the existing model terms, or by introducing modifications and, where necessary, novel physiological mechanisms. The resulting refined model is then parameterised using data from both the baseline and following weight stabilisation from the Yoyo study, and the values compared.

The study design and work-flow is visualised in Fig 1 and detailed below. In summary, three existing models of postprandial adipose tissue glucose, fatty acid, and triglyceride dynamics were evaluated using arteriovenous measurements across the abdominal subcutaneous adipose tissue. Subsequently, a refined model was constructed by either selecting the best fitting model term of the three existing models or, in cases where none of the existing models allowed for an adequate fit to the data, new or modified physiological mechanisms were introduced. Finally, model parameters were estimated before and after weight loss in the Yoyo study [17] and compared, taking their confidence intervals into account.

### Yoyo study and A-V measurements

The Yoyo study is a dietary intervention study designed to investigate the effect of the rate of weight loss (fast or slow) on subsequent weight regain [18]. Sixty-one overweight (BMI > 25kg/m^2^) and obese (BMI > 30kg/m^2^) individuals (BMI 28-35 kg/m^2^) were randomly assigned to one of two diet interventions designed to achieve a weight loss of approximately 10%, a low calorie diet of 1250 kcal/day over twelve weeks (slow weight loss) or a very low calorie diet of 500 kcal/day for five weeks (rapid weight loss). Following the diet intervention all participants underwent a four week weight stabilisation period with a diet based on the energy requirements of each participant. As part of this study, sixteen individuals underwent a high fat mixed-meal challenge test (milk shake) containing 100 mg of [U-^13^C] palmitate tracer at baseline and following the weight stabilisation period [17]. Fasting samples were collected at -30 and 0 minutes. The meal was consumed at 0 minutes, participants were asked to consume the shake within 10 minutes, and samples were collected at 60, 120, 180, 240, 300 minutes postprandially from an arterialised dorsal hand vein and the superficial epigastric vein draining the abdominal subcutaneous adipose tissue. Abdominal subcutaneous adipose tissue blood flow was measured using the ^133^Xe washout technique [19, 20].

### Ethics statement

All subjects gave their written informed consent before participation in the Yoyo Study. The Yoyo Study was performed according to the Declaration of Helsinki and was approved by the Medical Ethics Committee of Maastricht University Medical Centre (METC 11-3-066) with the approval number NL38099.068.11.

### Calculations

Individual metabolite fluxes across the abdominal subcutaneous adipose tissue were computed for NEFA, triglyceride, glucose, and glycerol by multiplying the arterio-venous plasma concentration difference of each measured metabolite by the adipose tissue blood flow [17].

Plasma tracer concentrations in NEFA and TG fractions were computed as described in the original study [17]. The rate of fractional spill-over of NEFA derived from LPL lipolysis of chylomicron triglyceride was calculated as one minus the rate of fractional extraction as described by Bickerton et al. [5]

Two indices of insulin resistance were calculated using the arterial metabolite measurements. HOMA-IR is a measure of whole body insulin resistance, and is calculated as fasting plasma glucose(mmol/l) times the fasting plasma insulin (*μ* U/ml) divided by 22.4 [21]. Several studied have evaluated appropriate HOMA-IR cutt-off values for determining insulin resistance, with identified cut-off calues ranging from 1.85 to 2.01 depending on the study population [22]. ADIPO-IR is a surrogate measure of adipose tissue specific insulin resistance and is calculated as fasting plasma NEFA concentration (mmol/l) times the fasting plasma insulin concentration (pmol/l) [23].

### Existing models

Models of postprandial lipid metabolism were assessed to identify models that described insulin mediated adipose tissue specific dynamics of glucose, triglyceride, NEFA, and/or glycerol. Three published models describing complementary aspects of adipose tissue metabolism were included in the analysis, namely the Jelic [11], Pratt [13], and Sips [10] models. The Pratt Model is a large, multi-compartmental, computational model describing the dynamics of several metabolite species including glucose, NEFA, glycerol, pyruvate, and both endogenous and dietary triglyceride in the fasting and postprandial states in plasma, skeletal muscle, adipose tissue, and liver [13]. The Jelic model is a two compartment, physiology-based, mathematical model describing NEFA dynamics across the plasma and a lumped interstitial adipose space in the postprandial state [11]. The Jelic model also accounts for two physiologically relevant delays in insulin signalling not present in the Pratt model. The model parameters for both the Jelic and Pratt models have been taken from literature and both models have been validated using oral glucose tolerance test (OGTT) and mix meal challenge test data in lean and abdominally obese individuals. The Sips model [10] is an extension of the Dalla-Man model [24] of postprandial glucose-insulin interplay to include NEFA kinetics. Unlike the previous two models, all parameters in the Sips model have been estimated from data. Where possible parameter values were maintained at the values provided for the lean healthy individual in the Dalla-Man model, as these parameters had been validated using gold standard triple-glucose tracer data [20]. The remaining 21 parameters were estimated from experimental data consisting of plasma measurements of several metabolites from individuals under various clamp conditions [25, 26] and also a frequently sampled oral glucose tolerance test (OGTT) and oral lipid tolerance test [10].

### Model evaluation and refinement

The three existing models (Jelic, Pratt, Sips) were decomposed into relevant subunits describing each measured metabolite’s dynamics across and within the adipose tissue using a so-called divide and conquer approach [27] (Fig 1, S1 Table). These model terms were then evaluated by comparison to the calculated metabolite flux from the A-V data (average *μ* = (*μ*_1_,…, *μ*_7_) and standard deviation *σ* = (*σ*_1_,…, *σ*_7_)) according to the following error function C(p):

$$\begin{array}{c} C\left(p\right)=\sum_{i=1}^{7}{\left(\frac{M\left(p,i(t_{i})\right)-\mu_{i}}{\sigma_{i}}\right)}^{2} \end{array}$$ (1)

Here *M*(*p*, *t*_*i*_) is the model prediction at time *t*_*i*_, *t* = (−30, 0, 60, 120, 180, 240, 300), for a parameter set *p*.

Optimal parameter sets for each term were obtained through non-linear regression, by finding the parameter set which best described the measured flux, minimising the error term C(p). Optimisation of parameters was performed using *lsqnonlin* (MATLAB 2014b, The MathWorks Inc., Natick, Massachusetts, United States) a local, gradient-based least square solver. The parameter values supplied in the original model studies were used as initial values for the parameter search. Parameterisation of the final, refined model for comparison of baseline and following weight stabilisation was performed using a combination of a global and local search algorithm. Controlled Random Search [28] with 250 randomly selected initial parameter sets was utilised to search the parameter space in order to provide a good initial value for lsqnonlin.

Terms describing the uptake and release of metabolites from tissues other than the adipose tissue, for example appearance from the gut and insulin secretion, are not described in this model. Available measured arterial concentrations of insulin, glucose, NEFA, triglyceride, and glycerol are supplied as dependent inputs to the model terms [29].

In the cases when the existing models were not capable of describing the experimentally estimated fluxes, underlying assumptions and model terms were evaluated based on existing biological knowledge and modified accordingly to provide an improved description of postprandial metabolite dynamics across the adipose tissue.

Additionally, all model terms were examined statistically, using the Akaike Information Criterion (AIC_c_) corrected for small sample sizes [30], to select the most parsimonious model that is both biologically sound and can adequately describe the measured flux data.

The above procedure was performed to evaluate terms describing the triglyceride, glucose, glycerol, and NEFA fluxes across the adipose tissue. Terms describing the fractional spill-over of NEFA by LPL lipolysis could also be assessed for the first time using the [U-^13^C] palmitate tracer data.

### Parameter identifiability

Model terms were also evaluated for the identifiability of parameters, as a primary objective of this study was to provide a model which could be parameterised by experimental data and could therefore be used to quantify adipose tissue metabolism from meal challenge test data. Identifiability of model parameters was evaluated using Profile Likelihood Analysis [31], whereby one parameter was varied iteratively from its optimal value and the remaining parameters were re-estimated. For an identifiable parameter the error measure *C*(*p*) would be expected to increase as the parameter value deviated from its optimum [31, 32]. 95% confidence intervals for the estimated parameter values have been estimated using the parameter value covariance matrix approximated using the Jacobian matrix provided as output of the lsqnonlin algorithm used in the parameter estimation procedure.

## Results

**Fig 2:**
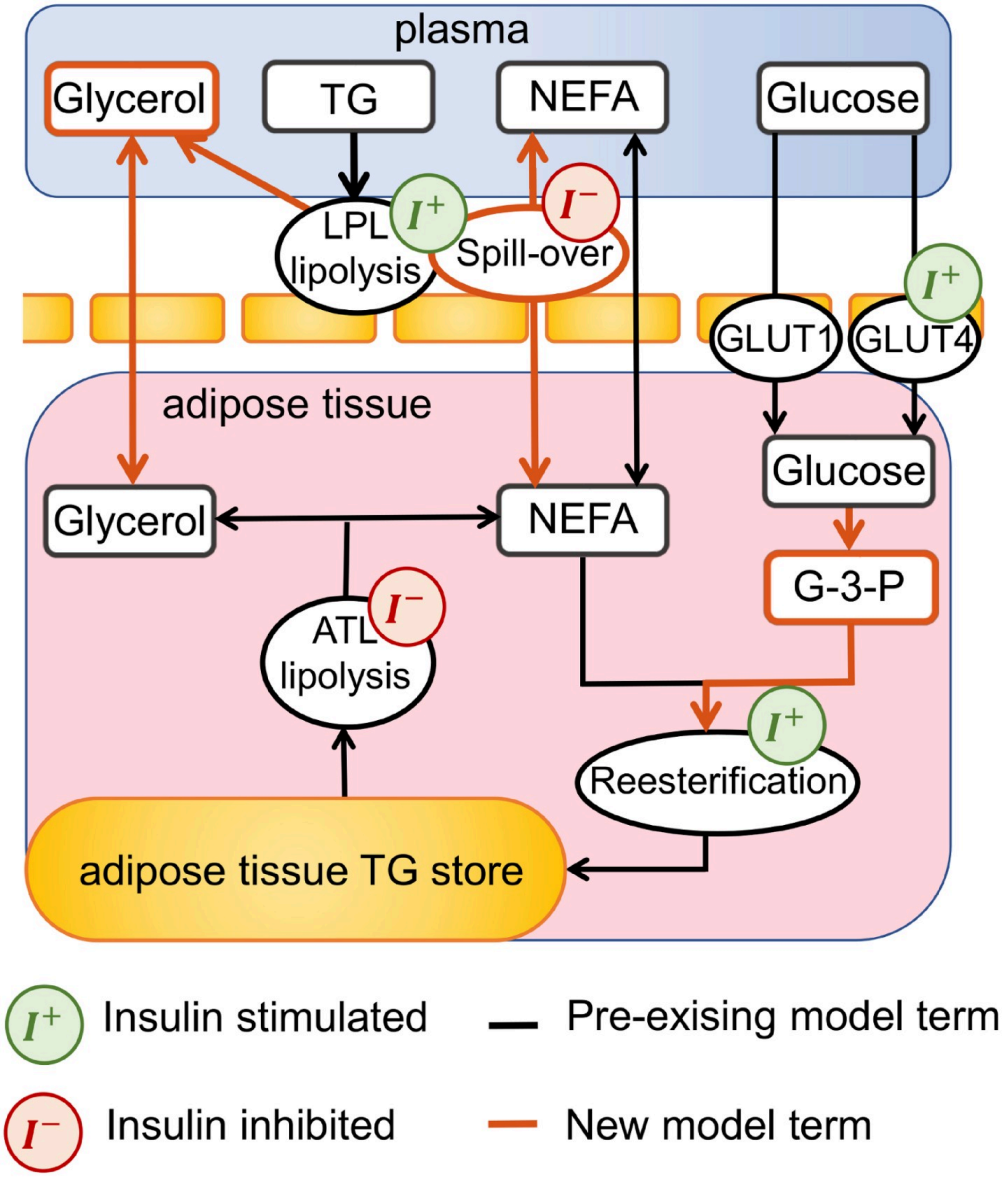
Structure of refined mathematical model of adipose tissue metabolism. The refined model of adipose tissue metabolism consists of a two compartmental model, describing dynamics between the plasma and a lumped interstitial adipose space. The insulin stimulated LPL lipolysis of circulating triglycerides releases glycerol and NEFA. The hydrolysed NEFA passes into the adipose space, with an insulin-dependent fraction spilling over into the plasma. The insulin inhibited lipolysis of the triglycerides stored within the adipose tissue releases NEFA and glycerol. It is assumed that this glycerol cannot be recycled within the adipose space and enters the plasma for transportation to the liver. Glucose passes into the adipose space at an insulin dependent and independent rate. Glucose is converted to G-3-P and provides the glycerol backbone necessary for re-esterification of NEFA for storage as triglyceride within the adipose space. Novel model terms, introduced in this analysis, are shown in red, existing models are shown in black. Reactions that are stimulated by insulin are depicted with a green insulin symbol. Reactions that are inhibited by insulin are shown with a red insulin symbol.

The three existing models of postprandial adipose tissue metabolism implemented in this study were not capable of describing the calculated postprandial adipose tissue metabolite fluxes. Consequently, a refined model of adipose tissue postprandial metabolism was constructed. The refined model is formulated as a two compartment model consisting of a blood plasma compartment and a lumped interstitial adipose compartment reflecting the sampling method used in the arteriovenous data (Fig 2). In the following, we show the results of the model evaluation and refinement separately for each measured metabolite using the adipose tissue flux data before the weight loss intervention.

### Triglyceride flux

Circulating plasma triglyceride is hydrolysed by LPL at the endothelial wall releasing NEFA and glycerol. The Pratt model describes this reaction as being proportional to the concentration of triglyceride present in circulating lipoproteins (both chylomicron and VLDL) and occurring at a basal and insulin stimulated rate using linear terms [13], with the plasma insulin concentration having an instantaneous effect. The Jelic model accounts for the saturation of enzyme mediated LPL lipolysis using Michaelis-Menten kinetics, with the rate of hydrolysis of circulating triglyceride being dependent on the plasma triglyceride concentration and stimulated by a delayed insulin effect [11]. The transcription of LPL and subsequent secretion of the LPL protein to the endothelial wall are known to be stimulated by insulin, however, these processes take some time [4]. As a result, the Jelic model introduced a three-fold insulin delay term, with the time delay parameter set to 240 mins [11]. The Sips model makes use of the Jelic model term and parameters [10].

**Fig 3:**
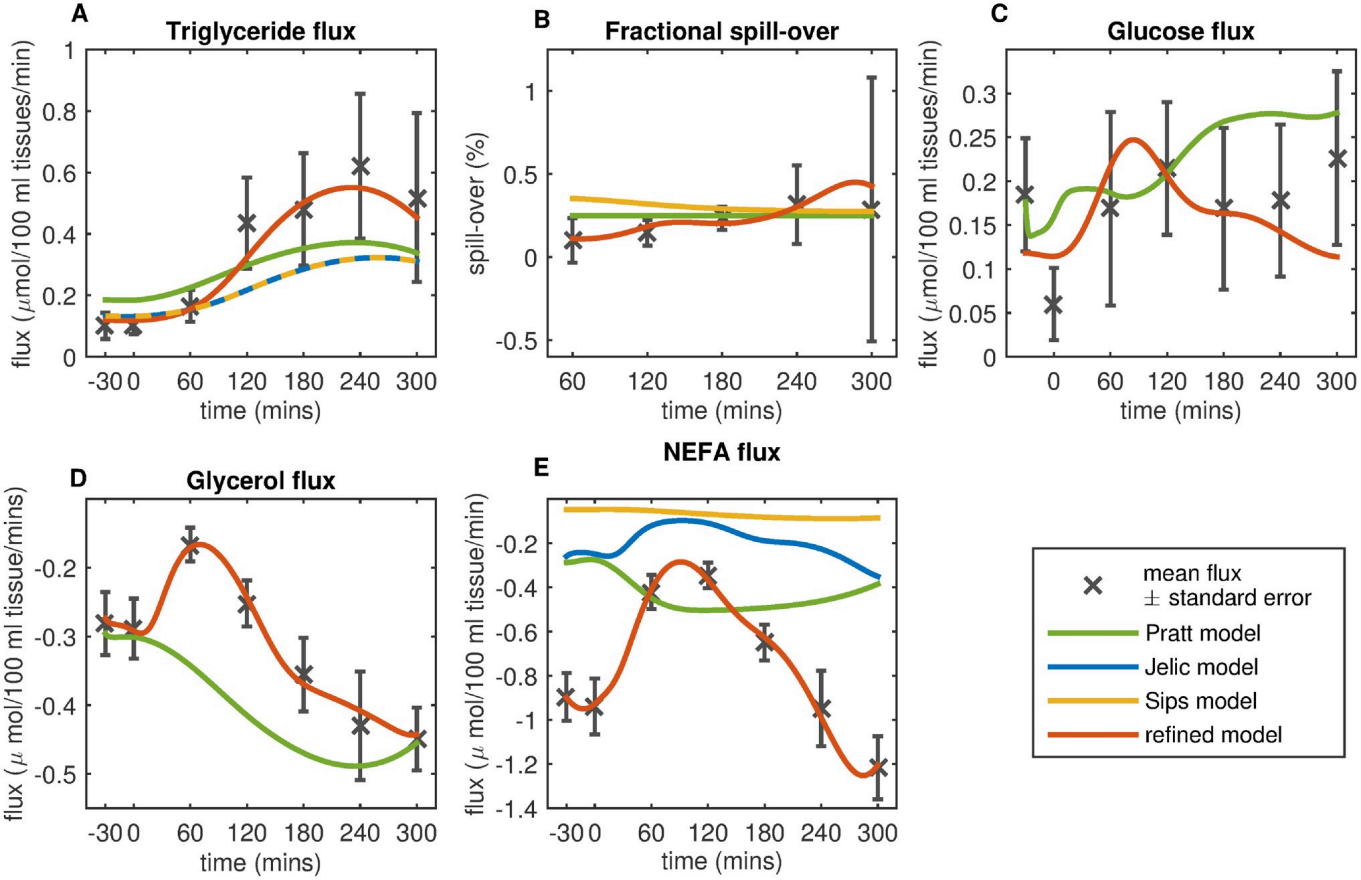
Comparison of the Jelic, Pratt, Sips, and refined model fit to baseline flux data from the Yoyo study. The fit of the refined model to the baseline adipose tissue flux data from the Yoyo Study is shown along with the terms from the Jelic, Pratt, and Sips models simulated using the parameter values provided in the respective publications. (A) Model terms from the Pratt (green), Jelic (blue), Sips (yellow), and refined model (red) describing the postprandial LPL mediated lipolysis of circulating triglycerides are shown. (B) Terms describing the fractional spill over of LPL derived NEFA from the Pratt, Sips, and refined adipose tissuse models are shown. The mean fractional spill over values, calculated using the postprandial palimate [U-^13^C] tracer included in the meal (black crosses ± standard error of mean). (C) Model terms describing the uptake of glucose into the adipose tissue from the Pratt and refined model are shown. (D) Depicts terms describing the postprandial efflux of glucose from the Pratt and refined models. (E) Model terms describing the efflux NEFA from the abdominal subcutaneous adipose tissue from the Pratt, Sips, Jelic, and refined models are shown. Metabolite fluxes are calculated as the arteriovenous difference in a metabolite across the adipose tissue multiplied by the rate of postprandial adipose tissue blood flow. The mean calculated adipose tissue fluxes are shown with the black crosses ± the standard error of the mean. Negative flux values indicate a net release of the metabolite from the adipose space, positive values indicate a net uptake.

Using the parameter values as specified in the original publications, neither the Jelic nor Pratt models are capable of describing the mean measured triglyceride flux across the adipose tissue (Fig 3A). Allowing the parameters to be estimated from the data, the Jelic model provides an improved fit. A more detailed investigation demonstrated that the insulin delay is the essential component missing in the Pratt model (S2 and S3 Figs). As a result, the optimised triglyceride flux term ([*TG*]_*flux*_) shown below was derived, making use of the insulin dependent linear approximation from the Pratt model while introducing the insulin delay term of the Jelic model.

$$\begin{array}{c} TG_{flux}=K_{ad}\left[{TG}_{art}\right]\left[I_{\text{LPL}}\right] \end{array}$$ (2)

**Fig 4:**
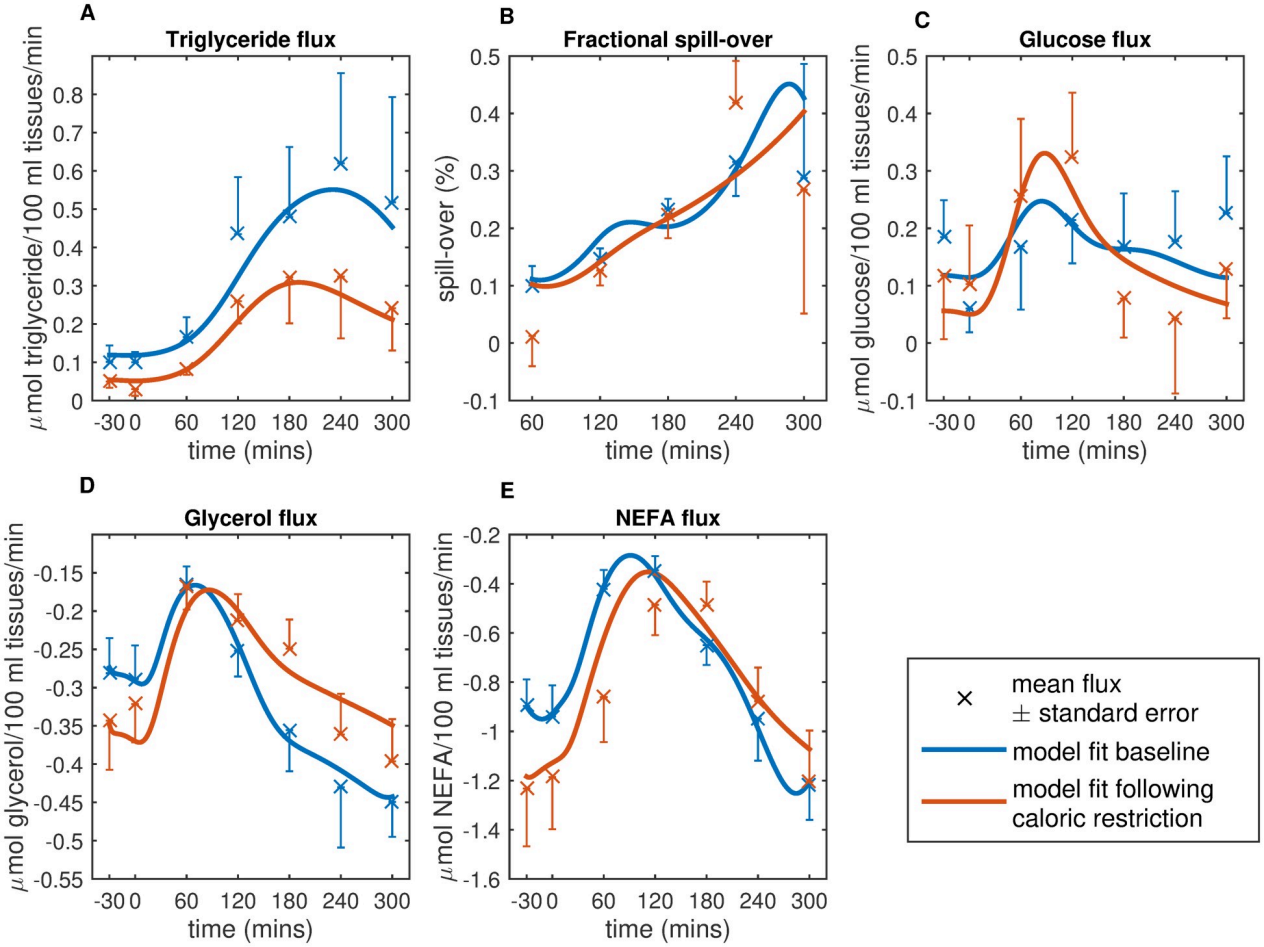
Fitting of the refined model to adipose tissue flux data measured at baseline and following caloric restriction from the Yoyo study. The refined model was fit to postprandial adipose tissue flux measurements of (A) LPL mediated lipolysis of circulating triglycerides, (B) the fractional spill over of LPL derived NEFA, (C) infflux of glucose, and efflux of both (D) glycerol, and (E) NEFA to the adipose tissue at baseline and following a period of caloric restriction. Blue crosses indicate the mean adipose tissue flux at baseline, the red crosses show the mean flux following caloric restriction, error bars indicate the standard error of the respective means. Refined model prediction at baseline is shown in blue, and the model fit following the diet intervention is shown in red. Negative flux values indicate a net release of the metabolite from the adipose space, positive values indicate a net uptake.

Where *K*_*ad*_ is the rate parameter for LPL lipolysis, [*TG*_*art*_] is the arterial triglyceride concentration and [*I*_LPL_] is the delayed insulin signal, modelled with a three compartmental delay (Eq 12) with a time delay parameter *τ*_*LPL*_ also estimated from data.(Fig 4)

### Fractional spill-over

NEFA released by LPL lipolysis of circulating triglyceride is taken up by the adipose tissue. However, this process is inherently leaky with a proportion of the released NEFA spilling-over directly into the plasma [33, 34]. Arteriovenous studies combined with NEFA stable isotope tracers have previously demonstrated that the fractional spill-over of NEFA from LPL lipolysis increases later in the postprandial period [5, 33].

The Jelic model does not describe the spill-over of NEFA derived from LPL lipolysis [11]. The Pratt model describes fractional spill-over at a constant rate of 25% [13]. The Sips model describes the fractional spill-over of LPL derived NEFA as occurring at a basal and insulin inhibited rate, using the same delayed insulin signal as LPL lipolysis with a 240 min time delay parameter [10].

The Sips term could not describe the increasing postprandial fractional spill-over measured in the Yoyo Study data (Fig 3B). The 240 min insulin time delay served to over-damp the insulin signal. As a result, the insulin delay was removed. Further analysis of the Sips term with use of the Akaike Information Criterion led to the removal of the basal fractional spill-over rate, yielding the following optimised fractional spill-over term.

$$\begin{array}{c} \%\text{spill-over}=\frac{1}{100}(D_{\text{spill}}\frac{I_{B}}{\left[I_{art}\right]}) \end{array}$$ (3)

Here *I*_*B*_ is the basal plasma insulin concentration, [*I*_*art*_] the arterial insulin concentration and *D*_spill_ an estimated constant (Fig 4B).

### Glucose flux

Glucose enters the adipose tissue along the concentration gradient facilitated by insulin dependent (GLUT4) and insulin independent (GLUT1) transporters [35]. Within the adipocyte, glucose is quickly converted to glucose-6-phosphate (G-6-P) which serves to trap the glucose within the adipocyte. Glucose is the primary source of the glyceraldehyde-3-phosphate (G-3-P) backbone needed for re-esterification of adipocyte NEFA [36].

Both the Pratt [13] and the Sips model [10] describes the uptake of glucose by the adipose tissue as occurring at an insulin dependent and independent rate. However, the Pratt model uses direct plasma insulin stimulation rather than accounting for a delay in the effect of insulin signalling which induces the translocation of GLUT4 transporters from the transport vesicles of the cell to the membrane. The Jelic model does not account for glucose dynamics.

With the introduction of a time delay in insulin stimulation the linear term from the Pratt model was capable of describing the glucose flux. In addition, it was necessary to explicitly account for the conversion of glucose to G-6-P in order to remedy an erroneous prediction of a postprandial glucose efflux from the interstitial adipose space (S3C Fig).

$$\begin{array}{c} G_{flux}=-GLUT1\left[G_{art}\right]-GLUT4\left[G_{art}\right]\left[I_{AT}\right] \end{array}$$ (4)

Where [*G*_*art*_] is the arterial glucose concentration, [*I*_*AT*_] is the adipose tissue delayed insulin signal described using a threefold delay with a time delay parameter *τ*_*AT*_(Eq 12), GLUT1 and GLUT4 are constants estimated from the data (Fig 4C).

### Glycerol flux

Glycerol is released into the plasma by LPL lipolysis of circulating triglyceride at the endothelial wall [35]. Glycerol is also released by lipolysis of triglyceride stored within the adipose tissue (denoted as ATL lipolysis [37]). It is commonly assumed tht due to the inactivity of glycerol kinase in the adipose tissue [38], all glycerol released by ATL lipolysis enters the plasma for transportation to the liver. Postprandial glycerol dynamics are not accounted for in the Jelic [11] nor Sips models [10]. The Pratt model accounts for the release of glycerol by ATL lipolysis within the adipose tissue which appears directly in the liver compartment [13]. Glycerol release into plasma by LPL lipolysis is not described in this model.

**Fig 5:**
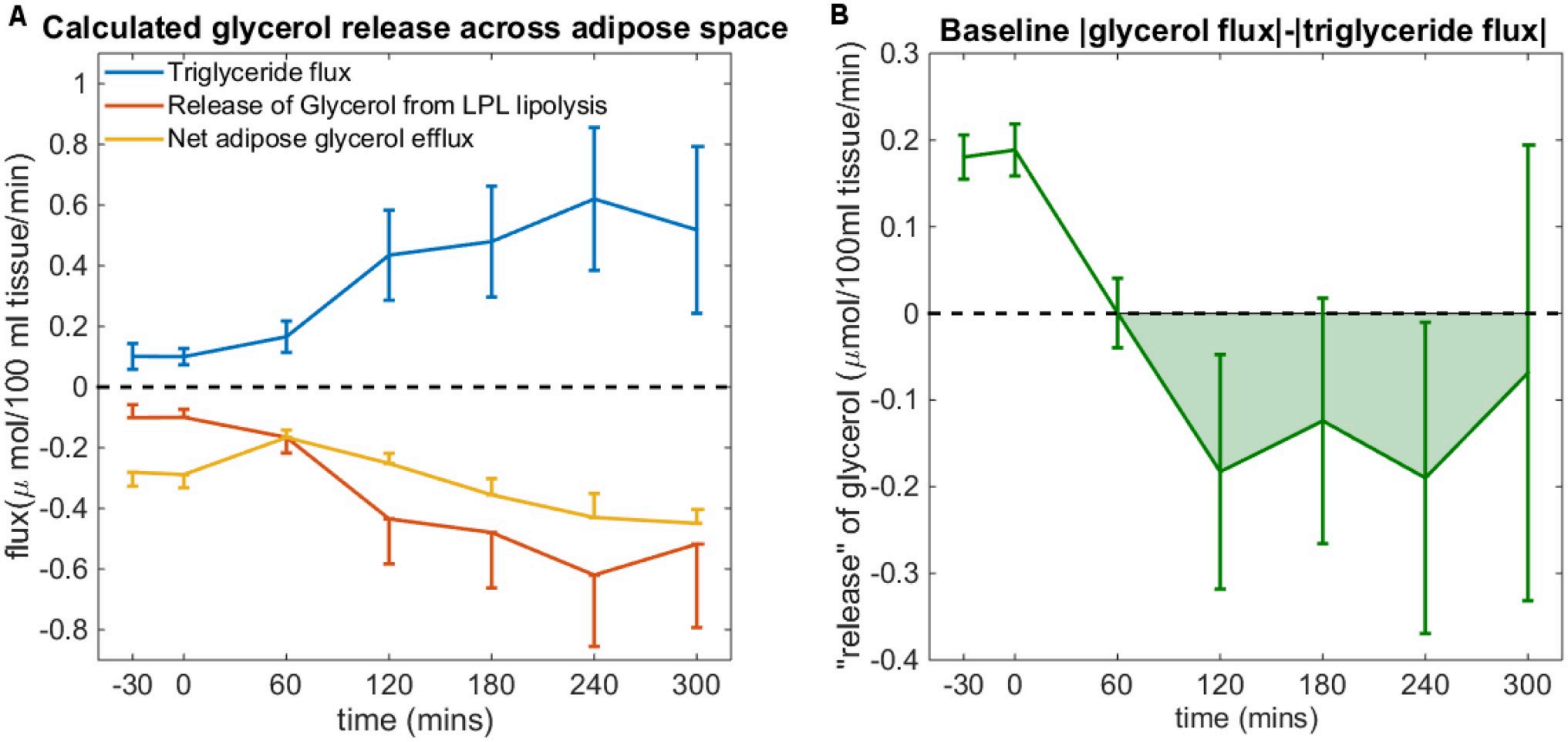
Postprandial uptake of glycerol by adipose space. A) Mean postprandial triglyceride influx (blue) (equivalent to the release of glycerol by LPL lipolysis (red)) and total glycerol efflux (yellow) across the abdominal subcutaneous adipose tissue at baseline in the Yoyo study (± standard error of the mean). B) Mean (± standard error of the mean) of glycerol flux minus triglyceride flux in postprandial phase measured at baseline in the Yoyo study indicating a net uptake of glycerol by the abdominal subcutaneous adipose tissue in the postprandial condition (green shaded region). This is in contradiction to the commonly held assumption that the glycerol efflux equals the direct sum of glycerol released by LPL lipolysis of circulating triglyceride and glycerol released by ATL lipolysis within the adipose tissue.

Analysis of the postprandial glycerol and triglyceride fluxes from the YoYo Study indicate the uptake of glycerol into the interstitial adipose space in the postprandial state (Fig 5). As a result, the Pratt glycerol term was extended to account for the concentration gradient diffusion of plasma glycerol into and from the interstitial adipose compartment.

$$GLY_{flux}=K_{ad}\left[{TG}_{art}\right]\left[I_{LPL}\right]+p_{GLY}\left(\left[{GLY}_{AT}\right]-\left(\left[{GLY}_{art}\right]+K_{ad}\left[TG_{art}\right]\left[I_{LPL}\right]\right)\right)$$ (5)

$$\frac{d[{GLY}_{AT}]}{dt}=-p_{GLY}\left(\left[{GLY}_{AT}\right]-\left[{GLY}_{art}\right]+K_{ad}\left[TG_{art}\right]\left[I_{LPL}\right]\right)+B_{ATL}+\frac{{ATL}_{max}}{1+\frac{\left[I_{AT}\right]}{K_{ATL}}}$$ (6)

Where ATL lipolysis is described as occurring at a basal (*B*_*ATL*_) and saturable insulin inhibited rate using terms from the Jelic model (*ATL*_*max*_, *K*_*ATL*_) [11]. Where [*I*_*AT*_] is the delayed interstitial adipose compartment insulin signal as described for the glucose flux above. (Figs 3D and 4D)

### NEFA flux

In the fasting state NEFA is released by the lipolysis of stored triglyceride within the adipocyte and passes into the plasma for delivery to other tissues. The lipolysis of stored triglyceride is inhibited by increasing insulin concentration in the postprandial state [39, 40] which also stimulates the rate of re-esterification of free NEFA within the adipocyte for storage as triglyceride [41]. In addition, insulin stimulates the LPL lipolysis of circulating triglyceride, removing excess dietary triglyceride from the plasma. While the majority of NEFA released by LPL lipolysis passes into the adipocyte for re-esterification a fraction spills-over directly into the plasma [33, 34].

Terms describing the fractional spill-over, LPL and ATL lipolysis have previously been derived. Terms describing the rate of re-esterification were compared using the Yoyo study NEFA flux. The Pratt model describes the rate of re-esterification as a linear term, directly stimulated by plasma insulin and adipose glucose concentration [13]. The Jelic model uses saturable kinetics coupled with a delayed insulin stimulation [11]. Both models used passive diffusion of NEFA between the plasma and interstitial adipose compartment. The Sips model does not explicitly describe the adipose compartment, instead using a linear term which accounts for net lipolysis and re-esterification of NEFA within the adipose tissue [10].

While the Jelic term could produce a good describtion of the NEFA flux when fit to the data (S2 Fig), it assumed an unlimited supply of G-3-P and required the estimation of five parameters from the data (S1 Table). The linear re-esterification term of the Pratt model was modified to include the delayed interstitial adipose compartment insulin signal. In addition, terms explicitly accounting for the production of the G-3-P backbone necessary for re-esterification from glucose taken into the adipose tissue were introduced, providing a sufficient description of the measured postprandial NEFA flux (Figs 3E and 4E).

$${NEFA}_{flux}=\frac{3}{100}(D_{\text{spill}}\frac{I_{B}}{\left[I_{art}\right]})K_{ad}\left[{TG}_{art}\right]\left[I_{\text{LPL}}\right]-p_{\text{NEFA}}(\left[{\text{NEFA}}_{PL}\right]-\left[{\text{NEFA}}_{AT}\right])$$ (7)

$$\begin{array}{cc} \frac{d[{NEFA}_{AT}]}{dt} & =\frac{3}{100}(1-D_{\text{spill}}\frac{I_{B}}{\left[I_{art}\right]})K_{ad}\left[{TG}_{art}\right]\left[I_{\text{LPL}}\right]+p_{\text{NEFA}}(\left[{\text{NEFA}}_{PL}\right]-\left[{\text{NEFA}}_{AT}\right])\\ & \;+3(B_{ATL}+\frac{{ATL}_{max}}{1+\frac{\left[I_{AT}\right]}{K_{ATL}}})-3(K_{\text{reester}}\left[I_{AT}\right]\left[{\text{NEFA}}_{AT}\right]\left[\text{G}-3-{\text{P}}_{AT}\right]) \end{array}$$ (8)

With $\left[{\text{NEFA}}_{PL}\right]=\left[{\text{NEFA}}_{art}\right]+\frac{3}{100}(D_{\text{spill}}\frac{I_{B}}{\left[I_{art}\right]})K_{ad}\left[{TG}_{art}\right]\left[I_{\text{LPL}}\right]$.

Where the adipose tissue concentration of G-3-P ([G-3-P_*AT*_]) calculated using the following equations.

$$\frac{d[\text{G}-3-{\text{P}}_{AT}]}{dt}=\left[\text{G}-3-{\text{P}}_{pro}\right]-K_{\text{reester}}\left[I_{AT}\right]\left[{\text{NEFA}}_{AT}\right]\left[\text{G}-3-{\text{P}}_{AT}\right]$$ (9)

$$\frac{d[\text{G}-6-\text{P}]}{dt}=\frac{1}{\tau_{\text{G}-3-\text{P}}}(2{\text{frac}}_{\text{use}}\left(GLUT1\left[G_{art}\right]+GLUT4\left[G_{art}\right]\left[I_{AT}\right]\right)-\left[\text{G}-6-\text{P}\right])$$ (10)

$$\frac{d[\text{G}-3-{\text{P}}_{pro}]}{dt}=\frac{1}{\tau_{\text{G}-3-\text{P}}}(\left[\text{G}-6-\text{P}\right]-\left[\text{G}-3-{\text{P}}_{pro}\right])$$ (11)

Re-esterification occurs at an insulin stimulated linear rate *K*_reester_ dependent on the available concentrations of G-3-P and NEFA. A portion of the glucose taken up by the adipose space (frac_use_) is converted to G-6-P and then to adipose tissue G-3-P ([G-3-P_*AT*_]) which will be available for use in re-esterification, the remaining glucose leaves the system for use by other cellular functions which are not explicitly described here. The production of adipose tissue G-3-P is described by the term [G-3-P_*pro*_] with a two compartmental delay governed by the delay constant *τ*_G-3-P_.

### Insulin delays

Insulin stimulates the translocation, secretion, and in some cases transcription of several enzymes and transport proteins involved in adipose tissue metabolism. These processes occur over time spans of several minutes to several hours. The effectiveness of the measured plasma insulin signal may be further damped by the presence of insulin resistance, a condition where the responsiveness of tissues to plasma insulin is reduced. Rather than describing the full sequence of reactions involved the dampening of the plasma insulin signal is approximated using a three compartmental delay, as in the Jelic model [11].

$$\frac{d[I_{1}]}{dt}=\frac{1}{\tau }\left(\left[I_{art}\right]-\left[I_{1}\right]\right)$$ (12)

$$\frac{d[I_{2}]}{dt}=\frac{1}{\tau }\left(\left[I_{1}\right]-\left[I_{2}\right]\right)$$ (13)

$$\frac{d[I_{\text{delay}}]}{dt}=\frac{1}{\tau }\left(\left[I_{2}\right]-\left[I_{\text{delay}}\right]\right)$$ (14)

Where [*I*_*art*_] is the measured arterial insulin concentration and *τ* is the respective time delay parameter. Two insulin time delays are simulated, a long time delay for LPL lipolysis (*τ* = *τ*_LPL_) and a shorter interstitial adipose compartment time delay (*τ* = *τ*_*AT*_). The structure of the insulin delay terms are not altered from the original model [11]. However, the time delay parameters are not fixed, as in the Jelic and Sips models, and are estimated from the data.

### Application of adipose model—Quantification of caloric restriction

The resulting refined model was then used to quantify the effect of caloric restriction using meal challenge test data collected before and after a diet intervention as part of the Yoyo study.

#### Parameter values before and after caloric restriction

Parameter sets were estimated from data collected at baseline and after a weight stabilisation period following caloric restriction (Table 1, the complete parameter sets are provided in S2 Table). The rate parameters for both glycerol and NEFA concentration gradient based diffusion from the plasma to the adipose space (*P*_*GLY*_ and *P*_*NEFA*_) increase significantly following caloric restriction, indicating an increase in the rate of glycerol and NEFA transport in and out of the adipose tissue. A large, although not significant, decrease can be seen in the insulin delay parameter for LPL lipolysis of circulating triglyceride, *τ*_*LPL*_, (157 mins at baseline to 113 mins following weight stabilisation) which is not accompanied by a strong change in the value for *K*_*ad*_, the rate parameter for that term. In addition, there is a four minute decrease in the adipose tissue insulin delay parameter *τ*_*AT*_. The reduction in the insulin delay parameters indicates a less damped response to insulin signalling following caloric restriction, indicative of improved insulin sensitivity.

**Table 1:** Parameter values estimated for data collected at baseline and following caloric restriction.

Parameters	Role/Function	Baseline	Following caloric restriction
*K*_*ad*_	Linear kinetic parameter LPL lipolysis.	0.0096 (0.0068, 0.0213)	0.0087 (0.0057, 0.0117)
*τ*_*LPL*_	LPL insulin delay	156.92 (68.3, 245.5)	112.76 (27.05, 198.48)
*τ*_*AT*_	Adipose insulin delay.	21.19 (-16.28, 58.66)*	17.13 (-10.34, 44.61)*
*P*_*GLY*_	Rate parameter for uptake/release of glycerol.	0.249 (0.109, 0.389)	17.13 (0.283, 0.566)
*P*_*NEFA*_	Rate parameter for uptake/release of NEFA.	0.0444 (0.0316, 0.0571)	0.0803 (0.041, 0.13)

Parameter values estimated by fitting the refined model to the calculated adipose tissue metabolite fluxes at baseline and following weight stabilisation for a selection of parameters are shown, 95% confidence intervals for parameter estimates are displayed in parentheses below the estimated value. The complete set of parameter values can be found in S2 Table.

*All parameters are bound below by zero during parameter estimation.

#### Decomposition of glycerol and NEFA model predictions

**Fig 6:**
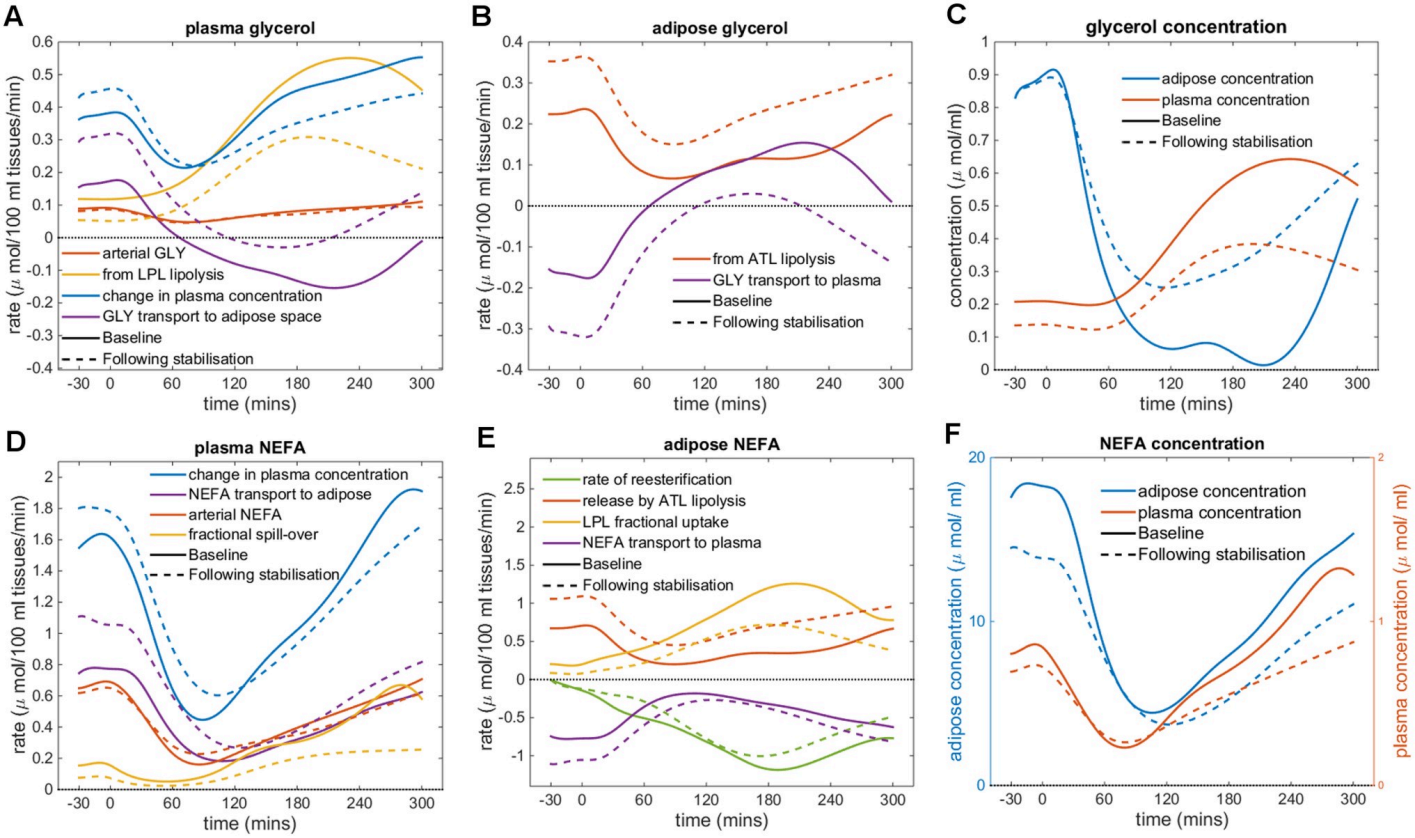
Decomposition of model glycerol and NEFA flux predictions into constituent reactions. Comparison of model predictions of rates of reactions contributing to the (A) glycerol flux in the plasma compartment, (B) rates of glycerol rate of reactions in adipose compartment, (C) plasma and adipose compartment glycerol concentrations, (D) NEFA flux in the plasma compartment, (E) rates of NEFA reaction in adipose compartment, and (F) plasma and adipose compartment NEFA concentrations. Solid lines represent baseline estimates and dashed lines represents estimates following weight stabilisation.

The estimated values for *P*_*GLY*_ and *P*_*NEFA*_ increased significantly following caloric restriction, but it remained unclear if the observed differences in the glycerol and NEFA fluxes following caloric restriction (Fig 4) could be entirely attributed to these increases in the rates of uptake and release of both metabolites. In Fig 6, our refined model’s predictions are used to decompose the calculated adipose glycerol and NEFA fluxes over the duration of the mixed meal challenge test into their constituent reactions. This allows for visualisation of how the rates of specific, unmeasured reactions change following caloric restriction. The model predicts an increase in the rate of ATL lipolysis within the adipose tissue following caloric restriction (Fig 6B and 6E, red lines), with a predicted 56% increase in the rate of lipolysis of stored triglyceride (0.229 *μ*mol/100 ml tissue/min at baseline to 0.359 *μ*mol/100 ml tissue/min following caloric restriction), resulting in the observed increase in efflux of both NEFA and glycerol from the adipose tissue in the fasting state (Fig 6A and 6D, purple lines). An increase in ATL lipolysis would be expected following such a period of caloric restriction as the triglyceride stores in the adipose tissue are hydrolysed to supply NEFA for use in other tissues. In the later postprandial phase, the lower concentrations of circulating triglyceride follow caloric restriction (total AUC 642.2 *μ*mol a baseline to just 463 *μ*mol following the diet intervention), results in the reduced efflux of glycerol and NEFA from LPL lipolysis after 120 minutes (Fig 6A and 6D, yellow lines).

Interestingly, the peak in the rate of LPL occurs approximately 40 mins earlier (230 mins at baseline versus 190 mins following caloric restriction) reflecting the estimated decrease in *τ*_*LPL*_ (Fig 6A and 6E, yellow lines). Note, there is no decrease in the peak time of measured arterial insulin (S4E Fig), however there is a decrease in both the fasting and postprandial plasma insulin concentrations (total AUC 6821.5 *μ*U of insulin at baseline to 5291.8 *μ*U following caloric restriction). Despite the decrease in circulating concentrations of insulin the postprandial inhibition of ATL lipolysis and stimulation of re-esterification by insulin occur at faster rates. The magnitude of slope describing the rate of postprandial inhibition of ATL lipolysis increases from 0.019 at baseline to 0.0023 following caloric restriction. Concurrently, the rate of stimulation of re-esterification increases from 0.0055 at baseline to 0.0078 following the diet intervention. These increases are indicative of improved responsiveness to circulating insulin, as reflected with the decrease in the estimated values for both insulin delay parameters.

#### Alternative measures of insulin resistance

Both whole body and adipose tissue specific insulin resistance were assessed using the HOMA-IR and ADIPO-IR indices respectively. HOMA-IR values decreased significantly from a mean values of 2.27 at baseline, which would be considered insulin resistant, to 1.51 following the diet intervention (p = 0.036). ADIPO-IR values also decreased significantly from 47.27 at baseline to 28.90 following caloric restriction (p = 0.006). Supporting the model prediction of an improvement in insulin sesntivity.

## Discussion

We present a refined mathematical model of postprandial adipose tissue insulin mediated dynamics of glucose, NEFA, triglyceride, and glycerol. Our refined model elucidates the strong effect of insulin on adipose tissue lipid metabolism, building on the work of Jelic [11], Pratt [13], and Sips [10], with all measured adipose tissue metabolite fluxes being stimulated or inhibited by insulin. Our model also introduces several novel terms. We explicitly account for the conversion of glucose to G-3-P, which serves to trap glucose within the adipose space. Thereby, counteracting the erroneous predictions of a postprandial efflux of glucose from the adipose compartment in the existing models due to glucose accumulation. We also introduce a term accounting for the concentration gradient dependent uptake and release of glycerol. This mechanism had been postulated by Coppack et al. in their 2005 model [42], but has not been accounted for in the three subsequently published models that we have compared. Moreover, inclusion of a [U-^13^C] palmitate stable isotope tracer in the ingested meal allowed us to estimate the postprandial fractional spill-over of LPL derived NEFA into the plasma. The corresponding model term has, to the best of our knowledge, not previously been validated using experimental data.

Arteriovenous measurements in the fasting and postprandial conditions across the abdominal subcutaneous adipose tissue were employed to evaluate three existing models of postprandial adipose tissue metabolism and the assumptions upon which they have been constructed. While the existing models included in this analysis perform well in describing dynamics between triglycerides, NEFA, and glucose at the whole-body level, none of the existing models could describe the adipose tissue specific fluxes well, leading to the construction of our refined model.

Methods such as area under the curve (AUC) are most often employed to compare time series of metabolite concentrations during challenge tests, with each metabolite evaluated independently of the others. However, AUC fails to capture the dynamic properties of the postprandial metabolite curves. It is possible for several different response curves to have the same AUC value. With computational models the dynamic responses of all measured metabolites are used to parameterise a model in a physiologically meaningful way. Consequently, computational models could also prove to be a powerful tool for the interpretation and quantification of dynamic time series of data, as in the case of meal challenge test data. Our refined model was applied to time series data collected before and after a weight loss intervention study [17] to investigate the effects of caloric restriction on adipose tissue metabolism. Comparison of parameter values estimated from data collected before and after caloric restriction indicated a significant increase in the rate coefficient for the concentration gradient based transport of both glycerol and NEFA in and out of the adipose compartment. Use of the model to decompose the glycerol and NEFA flux predictions into their constituent reactions predicted an increase in the rate of ATL lipolysis within the adipose tissue following caloric restriction, resulting in the measured increased efflux of NEFA from the adipose tissue in the fasting state. This would be expected following a period of caloric restriction as triglyceride stored in the adipose tissue is hydrolysed and the resulting NEFA is transported for use in other tissues. A large, but non-significant, decrease was observed in the insulin time delay parameter for LPL lipolysis of circulating triglyceride, *τ*_*LPL*_, from 157 minutes at baseline to 113 minutes following caloric restriction, the effect of which can be observed with the peak in the rate of LPL lipolysis occurring approximately 40 minutes earlier following weight stabilisation. This is accompanied by a non-significant four minute decrease in the general adipose tissue insulin delay *τ*_*AT*_. These reductions indicate a less damped response to insulin stimulation following the diet intervention, which is in line with previous studies reporting a reduction in whole body insulin resistance following prolonged caloric restriction [43]. Whole body insulin sensitivity for the sixteen individuals, measured using HOMA-IR, decreased significantly following caloric restriction, from a mean of 2.27 at baseline to 1.51 following the diet intervention (p = 0.036). In addition, adipose tissue specific insulin resistance, assessed using ADIPO-IR, decreased significantly following the diet intervention, further supporting our model’s prediction of improved insulin sensitivity following caloric restriction. It is also of note that the LPL time delay estimates are more than one, and close to two hours, shorter than the fixed 240 minutes time delay proposed by Jelic et al. [11] and subsequently used in the Sips model [10]. While changes could be observed in several other parameter values before and after caloric restriction, these differences were not significant. Previous analysis of the Yoyo study data using more traditional techniques (incremental area under the curve) found only a significant decrease in the fasting and postprandial triglyceride flux, however this was accompanied by reduced arterial triglyceride concentration [17]. Thus, we see the additional insights which can be gained through the fitting of computational models in the quantification of dynamic time series data [44].

Multiple arteriovenous studies have used the glycerol flux to infer information regarding rate of ATL lipolysis under the assumption that glycerol release by LPL lipolysis, equivalent to the instantaneous triglyceride flux, remains within the plasma, and due to the negligible activity of glycerol kinase within the adipose tissue [38], all glycerol relased by ATL lipolysis enters the plasma for transport to the liver. Thereby, the rate of ATL lipolysis can be estimted as the difference between the measured glycerol and triglyceride fluxes [45, 46]. However, analysis of the postprandial glycerol and triglyceride fluxes from the Yoyo Study indicates that there is an influx of glycerol into the interstitial adipose space in the postprandial state, in contradiction with this assumption (Fig 5). The introduction of a term accounting for the concentration gradient dependent uptake and release of glycerol by the adipose tissue, which was first proposed by Coppack et al. [41], allows the model to described the measured postprandial influx of glycerol. In addition, in order to achieve the best fit of the flux data, in particular the measured postprandial influx of glycerol from the plasma to the adipose tissue, it was necessary to introduce a sink term in adipose tissue glycerol which cannot be accounted for with current biological understanding. In future work it may be possible with the integration of adipose tissue specific measurements of other omics data to determine the cause of the glycerol disappearance.

In combination with arterio-venous measurements, the use of stable isotope tracers allow for the quantification of reactions that are not directly measurable, such as rates of appearance and turn over. The meal administered in the Yoyo study includes 100mg of [U-^13^C] palmitate stable isotope tracer which allows for the estimation of the rate of fractional spill-over of NEFA from LPL lipolysis of dietary triglycerides using the method as described by Bickerton et al. [5]. As the Yoyo study utilizes a single tracer we do not have the data to evaluate chylomicron and VLDL triglyceride separately. Therefore, we have grouped the terms and used the available measurements of fractional spill-over of chylomicron derived NEFA to be representative of fractional spill-over from total circulating triglyceride pool. Moreover, we assume the dynamics of palmitate is representative for the generic NEFA pool. Incorporation of additional fatty acid tracers under different challenge conditions would allow for the extension of our model to describe the complex systemic interplay between different lipoprotein classes and NEFA species. Inclusion of a palmitate stable isotope tracer in a meal has been shown to label dietary derived triglycerides and NEFA for approximately the first 120 to 180 minutes [5, 47]. Due to recycling of NEFA both within the adipose tissue and eventual incorporation of labelled NEFA into VLDL, estimates of fractional spill-over become less reliable beyond this time. This is reflected with the increases in the measured standard error of mean for later time points in part B of Fig 3. To this end, error measurements have been weighted by the standard deviation within the 16 individuals such that a time point with a larger standard deviation will have a lower priority when estimating parameter values.

In order to produce a model which could be parameterised from data, certain model terms were simplified from those presented in the original models, e.g. use of a linear term in place of Michaelis-Menten dynamics to describe LPL lipolysis of circulating triglycerides. A linear approximation is sufficient in this situation to describe this reaction, as saturation is not expected to occur given the measured arterial triglyceride concentrations within the Yoyo study. The use of Profile Likelihood Analysis indicates that six of the fourteen model parameters are identifiable given the data, with a further six having upper or lower bounds (S3 Fig). For future implementation of the model, fixation of parameter values for which reliable experimental estimates are available would improve the estimation of parameters of interest for the biological questions being asked. As with previous computational models of adipose tissue metabolism, we have considered whole body adipose tissue as one homogenous unit. While the arteriovenous sampling of the abdominal subcutaneous tissue technically restricts our model evaluations to a single adipose tissue depot, we believe that our model sufficiently captures postprandial adipose tissue dynamics given that the measured depot, the abdominal subcutaneous adipose tissue, is considered the primary site for the storage and release of NEFA into systemic circulation.

Arteriovenous measurements across other tissue depots, such as skeletal muscle, presents an opportunity to further evaluate and, where necessary, refine existing model terms describing contributions of other tissues to glucose and NEFA homeostasis, as in the case of adipose tissue metabolism in this study. In this study we present a work-flow where, through the use of dependent inputs in combination with a divide and conquer approach, the model evaluation procedure can be reduced to a non-linear regression problem, greatly reducing the computational time.

Given the association of ectopic fat deposition with the development of several metabolic disorders, such as Non-Alcoholic Fatty Liver Disease and Type 2 Diabetes, the study of the dysregulation of adipose tissue metabolism has garnered much attention in recent years. Our refined model is capable of simulating *in silico* the dynamics of human *in vivo* adipose tissue metabolism in both the fasting and postprandial state. Through the variation of model parameters or modulation of dependent inputs, such as the arterial insulin concentration, it is possible to simulate the dynamics in adipose tissue metabolism in response to different external stimuli and gain insight into potential sources of dysregulation in adipose metabolism. Furthermore, embedding our refined adipose tissue model into a larger whole body model, with terms describing the contributions of other tissues, would allow for the investigation how the model simulated dysregulation in adipose tissue metabolism would impact on the glucose and NEFA dynamics in other tissues and whole body metabolism.

In conclusion, we present a refined physiology-based computational model of adipose tissue metabolism which has been shown, using arteriovenous measurements across the abdominal subcutaneous adipose tissue, to outperform several existing models of adipose tissue metabolism. Our model elucidates the strong influence of insulin signalling on adipose tissue dynamics, particularly the cycling between storage and release of NEFA in the fasting and postprandial states. Application of our model to data collected before and after a diet intervention allows for quantification of the effect of caloric restriction on adipose tissue metabolism. Estimated parameter values indicate that the delays in insulin effectiveness in the system are not fixed, with the estimated time delays in insulin signalling not only differing from the values used in previous models, but also decreasing following the diet intervention suggesting an improvement in adipose tissue insulin sensitivity following caloric restriction.

## Supporting information

S1 Fig Results of Profile Likelihood Analysis for each parameter of the refined model. The parameter values estimated from the baseline data are shown with a red cross and the value of C(p) resulting from iteratively adjusting the parameter value and re-estimating the parameter values indicated by the blue line. A parabola with the parameter estimate at it’s base (as in the case of *K*_*ad*_, *τ*_*LPL*_, *D*_*spill*_, *τ*_*AT*_, *P*_*GLY*_, and *P*_*NEFA*_) indicates an identifiable parameter. Profile likelihood for several other parameters (GLUT1, GLUT4, *B*_*ATL*_, *ATL*_*max*_ and *τ*_*G*3*P*_) indicate the existence of an upper bound, these parameters have been bound below by zero in the parameter estimation procedure for physiologically relevant reasons. Similarly the parameter describing the fractional usage of glucose to for G-3-P production in re-esterification is bound above by one. Finally two parameters (*K*_*ATL*_, and *K*_*reester*_) appear to be practically non-identifiable. Given the product of *K*_*reester*_ and the model predicted concentration of G-3-P in the adipose tissue are equal to the maximum rate of G3P production it is unsurprising that the parameter *K*_*reester*_ in non-identifiable. Any change in the value of *K*_*reest*_ in compensated for by a corresponding change in the model predicted concentration of adipose G-3-P.
(TIF)

S2 Fig Results of fitting the Jelic, Pratt, Sips and refined models to the baseline adipose tissue flux data. Model simulation of available fluxes using parameter values estimated by fitting of each model to the the measured adipose (A) triglyceride flux, (B) fractional spill over of LPL derived NEFA, (C) glucose influx, (D) glycerol efflux, and (E) NEFA efflux are shown, Jelic (blue), Pratt (green), Sips (blue), and the refined model (red). Mean baseline calculated adipose tissue flux values ± the standard error of the mean from the Yoyo study are shown in black. The Jelic model is capable of producing quite a good fit of the triglyceride and NEFA fluxes. However, the Jelic model does not include terms to describe the frational spill over, glucose or glycerol fluxes. While the Pratt model can produce a qualitatively good fit of the glucose, NEFA, and glycerol fluxes it cannot produce a good fit of the triglyceride flux (A). The Jelic, Sips, and refined model descibed LPL lipolysis as being dependent on insulin, with a delayed insulin signal. The Pratt model also includes a term describing the stimulation of LPL lipolysis by insulin. However, it does not account for any delay in insulin signalling, with LPL lipolysis being directly stimulated by plasma insulin. The Pratt model also assumes that the contribution of insulin dependent lipolysis to the overall lipolysis of circulating triglyceride is neglible, consequently, the rate of LPL lipolysis is primarily determined by the circulating triglyceride concentration.
(TIF)

S3 Fig Increasing the influence of insulin dependent LPL lipolysis in the Pratt model. In the above figure we increase the weight of the contribution of insulin dependent LPL lipolysis, such that insulin dependent LPL lipolysis accounts for 1% (blue line) and 10% (yellow line) of the total adipose tissue triglyceride flux while maintaining the other parameters at the values provided in the original publication. As the Pratt model uses direct plasma insulin stimulation rather than accounting for delays in insulin signalling, as in the Jelic, Sips, and refined models, the model simulated triglyceride flux begins to peak too early under the influence of plasma insulin. The refined model makes use of the LPL lipolysis term from the Pratt model, but introduces the three compartmental delay from the Jelic and Sips models. With this delay in the insulin signalling, the refined model can produce a good fit to the triglyceride flux data at baseline and following weight stabilsation (red line).
(TIF)

S4 Fig Mesured arterial metabolite concentrations at baseline and following caloric restriction. Comparison of measured arterial concentrations of triglyceride, glucose, glycerol, NEFA, and insulin colected during consumption of a high fat mixed meal at baseline (blue) and after a period of weight stabilisation following prolonged caloric restriction (red). Mean values for the sixteen participants are shown, with error-bars indicating the standard error of the mean.
(TIF)

S1 Table Comparison of terms from the Jelic, Pratt, Sips, and refined adipose tissue models. Terms describing individual metabolite fluxes across the adipose tissue were extracted from the Jelic, Pratt, Sips, and refined model and compared.
(PDF)

S2 Table Parameter values estimated for data collected at baseline and following caloric restriction. Complete set of parameter values estimated by fitting the refined model to the calculated adipose tissue metabolite fluxes at baseline and following weight stabilisation. 95% confidence intervals for parameter estimates are displayed in parentheses below the estimated value. The coloured boxes indicate the model term, or terms, in which each parameter appears. *All parameters were bound below by zero during parameter estimation, however the method for calculating the confidence intervals assumes the confidence interval is symmertric about the estimated parameter values. † terms that decribe a fractional value were also bound above by 1 during parameter estimation.
(PDF)

S1 File Sensitivity analysis for refined model. (PDF)

S2 File Complete model equations. (PDF)

S3 File Matlab implementation of refined adipose tissue model. (ZIP)
