# Supplementary figures and images for: A computational model of postprandial adipose tissue lipid metabolism derived using human arteriovenous stable isotope tracer data
Source: PLoS Comput Biol. 2019 Oct 3;15(10):e1007400. doi: 10.1371/journal.pcbi.1007400 (PMC6890259; doi:10.1371/journal.pcbi.1007400)

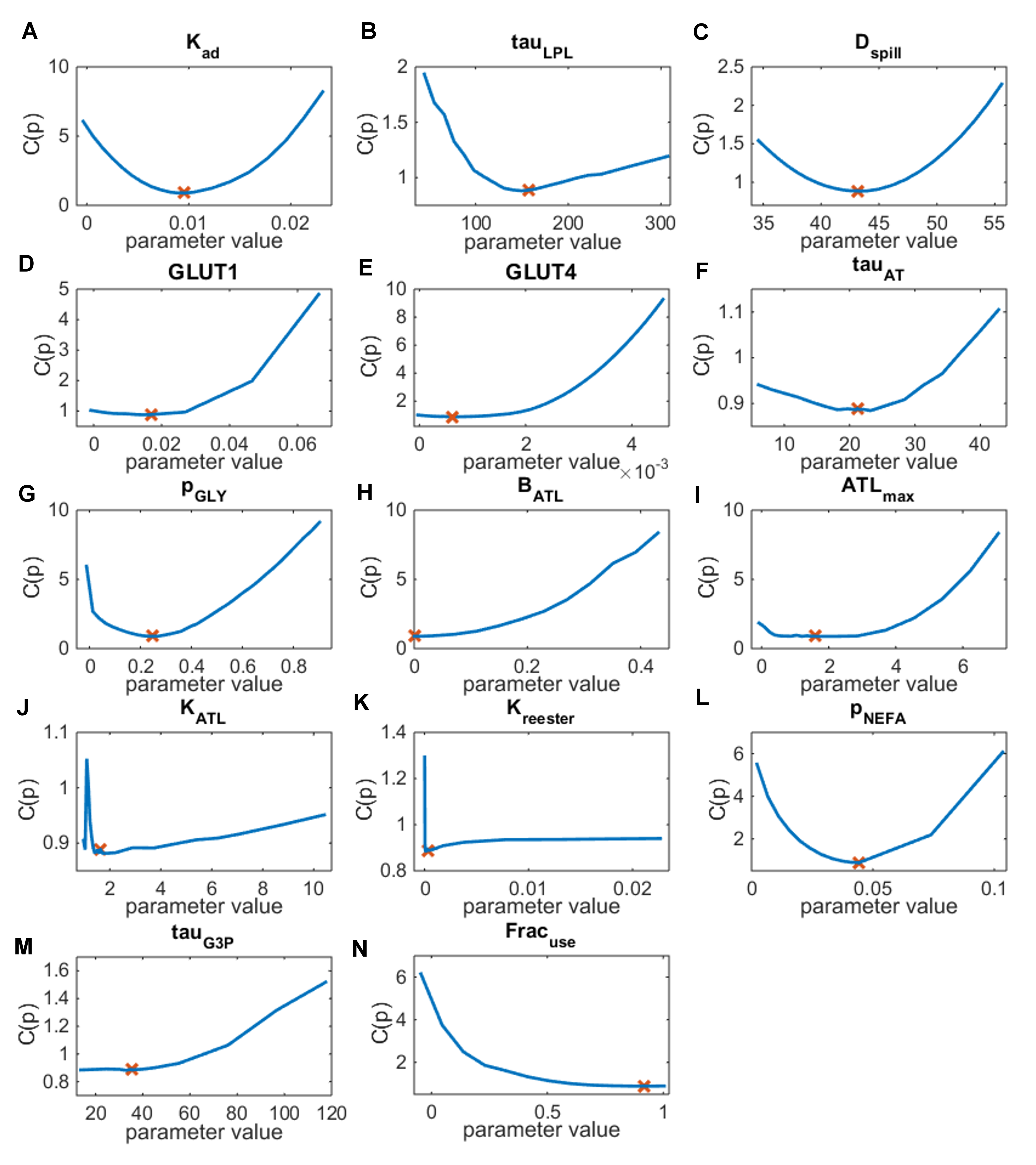

Supplement: S1 Fig — The parameter values estimated from the baseline data are shown with a red cross and the value of C(p) resulting from iteratively adjusting the parameter value and re-estimating the parameter values indicated by the blue line. A parabola with the parameter estimate at it’s base (as in the case of Kad, τLPL, Dspill, τAT, PGLY, and PNEFA) indicates an identifiable parameter. Profile likelihood for several other parameters (GLUT1, GLUT4, BATL, ATLmax and τG3P) indicate the existence of an upper bound, these parameters have been bound below by zero in the parameter estimation procedure for physiologically relevant reasons. Similarly the parameter describing the fractional usage of glucose to for G-3-P production in re-esterification is bound above by one. Finally two parameters (KATL, and Kreester) appear to be practically non-identifiable. Given the product of Kreester and the model predicted concentration of G-3-P in the adipose tissue are equal to the maximum rate of G3P production it is unsurprising that the parameter Kreester in non-identifiable. Any change in the value of Kreest in compensated for by a corresponding change in the model predicted concentration of adipose G-3-P. (TIF) [file pcbi.1007400.s001.tif]

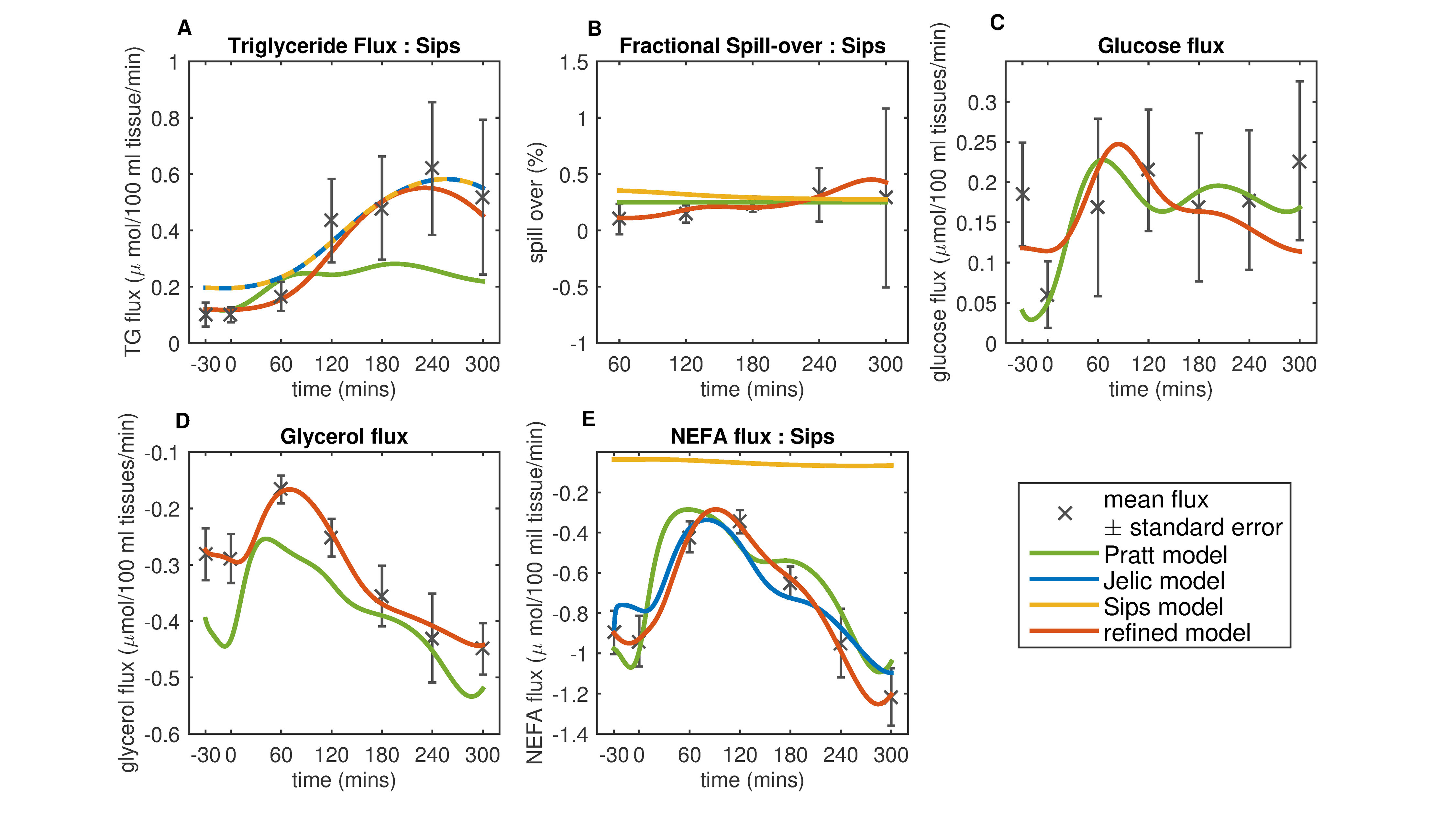

Supplement: S2 Fig — Model simulation of available fluxes using parameter values estimated by fitting of each model to the the measured adipose (A) triglyceride flux, (B) fractional spill over of LPL derived NEFA, (C) glucose influx, (D) glycerol efflux, and (E) NEFA efflux are shown, Jelic (blue), Pratt (green), Sips (blue), and the refined model (red). Mean baseline calculated adipose tissue flux values ± the standard error of the mean from the Yoyo study are shown in black. The Jelic model is capable of producing quite a good fit of the triglyceride and NEFA fluxes. However, the Jelic model does not include terms to describe the frational spill over, glucose or glycerol fluxes. While the Pratt model can produce a qualitatively good fit of the glucose, NEFA, and glycerol fluxes it cannot produce a good fit of the triglyceride flux (A). The Jelic, Sips, and refined model descibed LPL lipolysis as being dependent on insulin, with a delayed insulin signal. The Pratt model also includes a term describing the stimulation of LPL lipolysis by insulin. However, it does not account for any delay in insulin signalling, with LPL lipolysis being directly stimulated by plasma insulin. The Pratt model also assumes that the contribution of insulin dependent lipolysis to the overall lipolysis of circulating triglyceride is neglible, consequently, the rate of LPL lipolysis is primarily determined by the circulating triglyceride concentration. (TIF) [file pcbi.1007400.s002.tif]

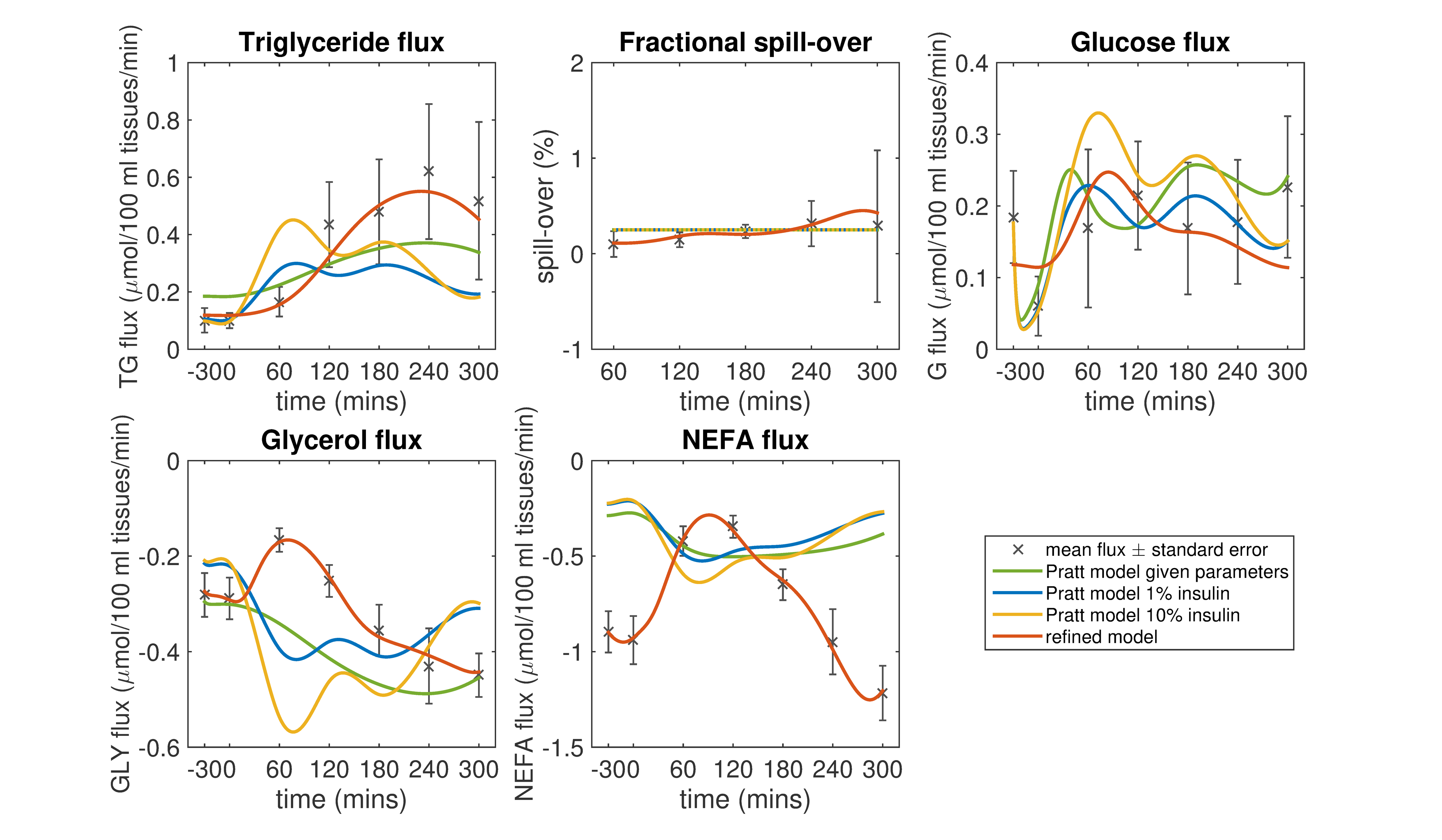

Supplement: S3 Fig — In the above figure we increase the weight of the contribution of insulin dependent LPL lipolysis, such that insulin dependent LPL lipolysis accounts for 1% (blue line) and 10% (yellow line) of the total adipose tissue triglyceride flux while maintaining the other parameters at the values provided in the original publication. As the Pratt model uses direct plasma insulin stimulation rather than accounting for delays in insulin signalling, as in the Jelic, Sips, and refined models, the model simulated triglyceride flux begins to peak too early under the influence of plasma insulin. The refined model makes use of the LPL lipolysis term from the Pratt model, but introduces the three compartmental delay from the Jelic and Sips models. With this delay in the insulin signalling, the refined model can produce a good fit to the triglyceride flux data at baseline and following weight stabilsation (red line). (TIF) [file pcbi.1007400.s003.tif]

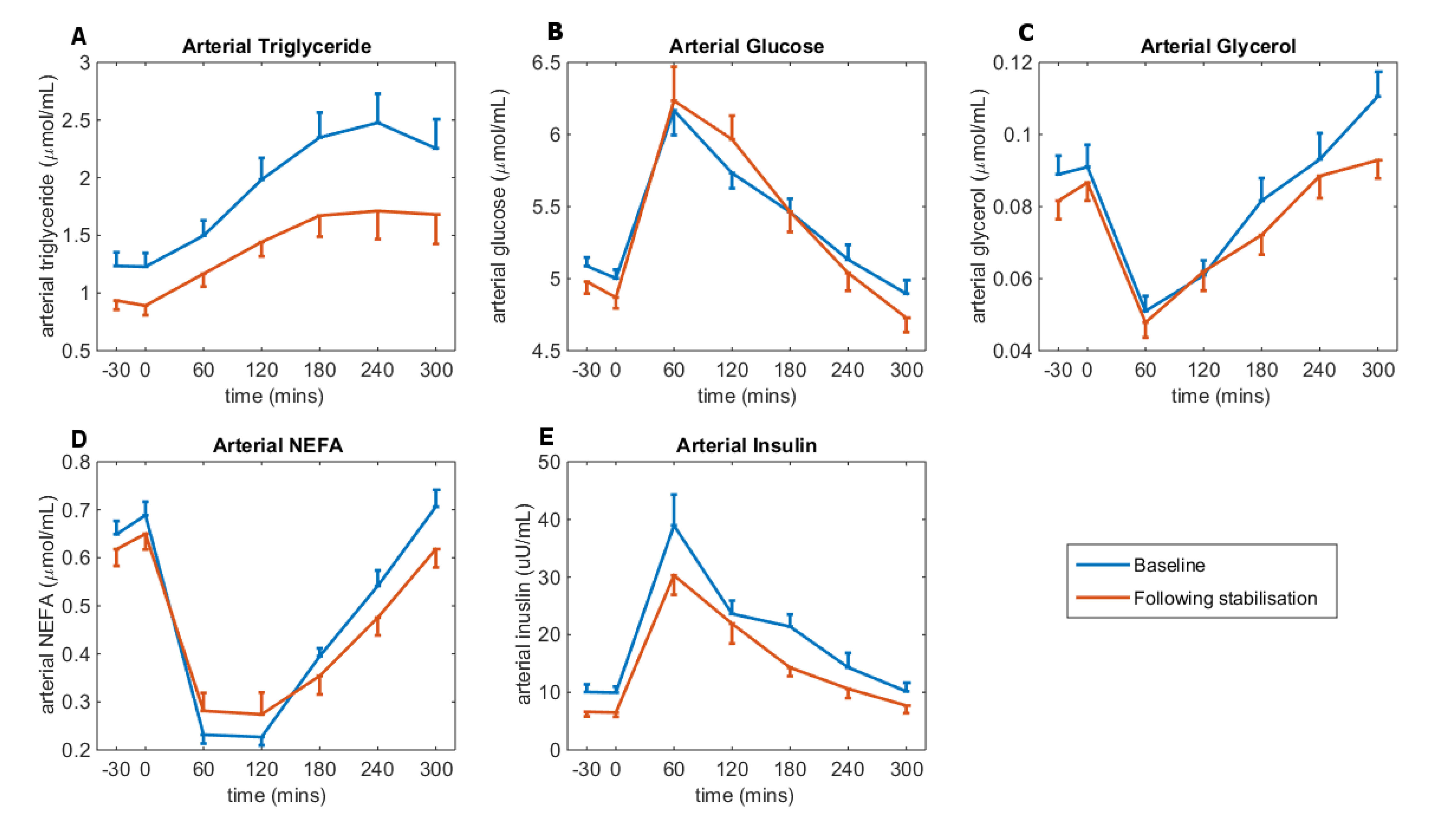

Supplement: S4 Fig — Comparison of measured arterial concentrations of triglyceride, glucose, glycerol, NEFA, and insulin colected during consumption of a high fat mixed meal at baseline (blue) and after a period of weight stabilisation following prolonged caloric restriction (red). Mean values for the sixteen participants are shown, with error-bars indicating the standard error of the mean. (TIF) [file pcbi.1007400.s004.tif]
