# Supplementary material for: A computational model of postprandial adipose tissue lipid metabolism derived using human arteriovenous stable isotope tracer data
Source: PLoS Comput Biol. 2019 Oct 3;15(10):e1007400. doi: 10.1371/journal.pcbi.1007400 (PMC6890259; doi:10.1371/journal.pcbi.1007400)
Supplement: S1 Table — Terms describing individual metabolite fluxes across the adipose tissue were extracted from the Jelic, Pratt, Sips, and refined model and compared. (PDF) [file pcbi.1007400.s005.pdf]

Supplementary Table S1: Comparison of terms from the Jelic, Pratt, Sips, and refined adipose tissue models.

Term	Jelic Model [1]	Pratt Model [2]	Sips Model [3]	Refined Model
LPL lipolysis	$= -\frac{LPL_{max}\cdot[TG_{PL}]}{K_{LPL}+[TG_{PL}]}\cdot\frac{P\cdot[I_{LPL}]}{K_P+[I_{LPL}]}$<br>• Accounts for enzyme kinetics.<br>• Insulin action is modelled with a third order delay. $[I_{LPL}]$ (240 min delay)	$= -K_a(1+K_{ai}[I_{PL}][TG_{PL}])$<br>• No enzyme kinetics. (linear dynamics)<br>• No insulin delay.<br>(plasma insulin stimulation $[I_{PL}]$)	$= -\frac{LPL_{max}\cdot[TG_{PL}]}{K_{LPL}+[TG_{PL}]}\cdot\frac{P\cdot[I_{LPL}]}{K_P+[I_{LPL}]}$<br>• As in Jelic model.	$= -k_{ad}[I_{LPL}][TG_{PL}]$<br>• No enzyme kinetics<br>(do not have data to accurately estimate parameters.)<br>• Introduces Jelic model insulin delay. $[I_{LPL}]$
ATL lipolysis	$= B_{ATL} + \frac{ATL_{max}}{1+\frac{[I_{AT}]}{K_{ATL}}}$<br>• Occurs at basal and saturable rate.<br>• Inhibited by delayed insulin $[I_{AT}]$	$= \frac{3\beta_t}{1+K_{Ft}[I_{PL}]^2}$<br>• Saturable rate.<br>• Inhibited by plasma insulin. $[I_{PL}]$<br>• Released NEFA appears directly in plasma compartment.	$= p_B([I_{d7}]-I_b)[NEFA]$<br>• Linear term governed by difference between delayed insulin $[I_{d7}]$ and basal insulin. I_b	$= B_{ATL} + \frac{ATL_{max}}{1+\frac{[I_{AT}]}{K_{ATL}}}$<br>• Occurs at basal and saturable rate.<br>• Inhibited by delayed insulin. $[I_{AT}]$
Re-esterification	$= -\frac{RST_{max}\cdot[NEFA_{AT}]}{K_{RST}+[NEFA_{AT}]}\cdot\frac{Q\cdot[I_{AT}]}{K_Q+[I_{AT}]}$<br>• Saturable rate.<br>• Stimulated by delayed insulin. $[I_{AT}]$<br>• Independent of glucose/G-3-P concentration.	$= -K_{aa}[I_{PL}][NEFA_{AT}][G_{AT}]$<br>• Linear rate.<br>• Stimulated by plasma insulin. $[I_{AT}]$<br>concentration.	Not present<br>• Re-esterification is not explicitly described. Coupled with term describing NEFA flux.	$= -3K_{reest}[I_{AT}][NEFA_{AT}][G-3-P_{AT}]$<br>• Linear rate.<br>• Stimulated by delayed insulin.$[I_{AT}]$<br>• Dependent on adipose G-3-P concentration.
Fractional spill-over	Not present<br>• All NEFA released by LPL lipolysis enters interstitial adipose compartment.	spill-over $= K_{lp}$<br>• Occurs at a constant rate $K_{lp} = 0.25$.	spill-over $= \frac{A_{\text{spill}}+B_{\text{spill}}\frac{I_b}{[I_{d6}]}}{100}$<br>• Insulin independent and delayed insulin $[I_{d6}]$ dependent term.	spill-over $= \frac{D_{\text{spill}}\frac{I_b}{[I_{PL}]}}{100}$<br>• Plasma insulin dependent. $[I_{PL}]$

Term	Jelic Model [1]	Pratt Model [2]	Sips Model [3]	Refined Model
Glucose flux	Not present<br>• Does not describe glucose dynamics.	$= -d_{BA}(1 + K_{ga}[I_{PL}])([G_{PL}] - [G_{AT}])$<br>• Occurs at plasma insulin dependent $[I_{PL}]$ and insulin independent rate.<br>• Rate dependent on relative concentration of adipose and plasma glucose.	$= -\frac{(K_{uid1}+K_{uid3}\frac{[I_{d3}]}{[NEFA_{d1}]})[G_t]}{K_{m,uid}+[G_t]}$<br>• No explict adipose glucose flux.<br>• Occurs at insulin and NEFA dependent and independent rate.<br>• Delayed insulin stimulation $[I_{d3}]$<br>• Delayed NEFA inhibition	$= -(GLUT_1 + GLUT_4[I_{AT}])[G_{PL}]$<br>• Occurs at insulin dependent and insulin independent rate.<br>• Assumes adipose glucose concentration is negligible.<br>$[NEFA_{d1}]$.• Delayed insulin stimulation
NEFA flux	$= -PP([\text{NEFA}_{PL}] - [\text{NEFA}_{AT}])$<br>• Concentration gradient dependent transport.<br>• Fractional spill-over not described.	$= -k_{na}[\text{NEFA}_{PL}] + \frac{3\beta_f}{1+K_{ft}[I_{PL}]^2}$<br>$+3K_a \cdot K_{lp}(1 + K_{ai}[I_{PL}])[TG_{PL}]$<br>• Linear term describing NEFA uptake. only.<br>• NEFA release by ATL lipolysis contributes directly to plasma NEFA.<br>• Fractional spill-over of LPL derived NEFA contributes to flux.	$= -(P_A + P_B([I_{D7}] - I_b))[\text{NEFA}_{PL}]$<br>$+\frac{spill}{V_{NEFA}}(\frac{LPL_{max}\cdot[TG_{PL}]}{K_{LPL}+[TG_{PL}]} \cdot \frac{P\cdot[I_{LPL}]}{K_P+[I_{LPL}]})$<br>• Linear term describing NEFA uptake.<br>• NEFA released by ATL lipolysis contributes directly to NEFA flux.<br>• Fractional spill-over of LPL derived NEFA contributes to flux.	$= -P_{NEFA}([\text{NEFA}_{PL}] - [\text{NEFA}_{AT}])$<br>$+3(\frac{D_{\text{spill}}\frac{I_b}{[I_{PL}]}}{100})K_{ad}[I_{LPL}][TG_{PL}]$<br>• Concentration gradient dependent transport.<br>• Fractional spill-over of LPL derived NEFA contributes to flux.
Glycerol flux	Not present<br>• There is no description of glycerol dynamics.	Not present<br>• Glycerol released by ATL lipolysis appears directly in the liver compartment.<br>• Glycerol released by LPL lipolysis not described.	Not present<br>• There is no description of glycerol dynamics.	$= -P_{GLY}([GLY_{AT}] - [GLY_{PL}])$<br>$+K_{ad}[I_{LPL}][TG_{PL}]$<br>• Concentration gradient dependent transport of glycerol.<br>• Glycerol released from LPL lipolysis contributes to flux.

Terms describing individual metabolite fluxes across the adipose tissue were extracted from the Jelic, Pratt, and Sips model and compared using the Yoyo study arterio-venous measurements. The resulting refined model terms are shown in the fifth column.

References:

1. Jelic K, Hallgreen C E, Colding-Jørgensen M. “A Model of NEFA Dynamics with Focus on the Postprandial State”, *Ann Biomed Eng* (2009) 37: 1897.
2. Pratt AC, Wattis JA, Salter AM. ”Mathematical modelling of hepatic lipid metabolism”, *Math Biosci* (2015) 262:167-181.
3. Sips FLP, Nyman E, Adiels M, Hilbers PAJ, Strålfors P, van Riel NAW, Gunnar Cedersund. “Model-Based Quantification of the Systemic Interplay between Glucose and Fatty Acids

in the Postprandial State." *PLoS ONE* (2015) 10(9): e0135665.
