# Supplementary material for: A computational model of postprandial adipose tissue lipid metabolism derived using human arteriovenous stable isotope tracer data
Source: PLoS Comput Biol. 2019 Oct 3;15(10):e1007400. doi: 10.1371/journal.pcbi.1007400 (PMC6890259; doi:10.1371/journal.pcbi.1007400)
Supplement: S2 Table — Complete set of parameter values estimated by fitting the refined model to the calculated adipose tissue metabolite fluxes at baseline and following weight stabilisation. 95% confidence intervals for parameter estimates are displayed in parentheses below the estimated value. The coloured boxes indicate the model term, or terms, in which each parameter appears. *All parameters were bound below by zero during parameter estimation, however the method for calculating the confidence intervals assumes the confidence interval is symmertric about the estimated parameter values. † terms that decribe a fractional value were also bound above by 1 during parameter estimation. (PDF) [file pcbi.1007400.s006.pdf]

Flux	Role/Function	Parameter	Baseline	Following caloric restriction
Triglyceride	Linear kinetic parameter.	K_{ad}	0.0096 (0.0068 , 0.0213)	0.0087 (0.0057 , 0.0117)
	LPL insulin delay	τ_{LPL}	156.92 (68.3 , 245.5)	112.76 (27.05 , 198.48)
Spill-over	Fractional spill-over	D_{spill}	43.17 (37.98 , 48.37)	46.91 (34.67 , 59.15)
Glucose	Insulin independent uptake	GLUT1	0.017 (-0.002 , 0.036)*	2.16 $\times 10^{-7}$ (-0.03,0.03) *
	Insulin dependent uptake.	GLUT4	6.106 $^{-4}$ (-4.02 $\times 10^{-4}$, 0.002)*	0.0017 (-2.72 $\times 10^{-4}$, 0.0037)*
	Adipose inuslin delay.	τ_{AT}	21.19 (-16.28 , 58.66)*	17.13 (-10.34 , 44.61)*
Glycerol	Rate parameter for uptake/release.	P_{GLY}	0.249 (0.109 , 0.389)	17.13 (0.283 , 0.566)
	Basal ATL lipolysis.	B_{ATL}	0.001 (-0.467 , 0.469)*	2.1 $\times 10^{-4}$ (-0.781 , 0.781) *
	V_{max} of enzyme mediated ATL lipolysis.	ATL_{max}	1.595 (-56.352 , 59.542)*	0.559 (-0.384 , 1.501)*
	K_{m} of enzyme mediated ATL lipolysis.	K_{ATL}	1.631 (-71.268 , 74.531)*	11.312 (-78.68 , 101.31)*
NEFA	Rate of re-esterification.	K_{reest}	3.019 $\times 10^{-4}$ (-9.079 $\times 10^{-4}$, 0.0015)*	0.011 (-0.0101 , 0.0122)*
	Rate parameter for uptake/release.	P_{NEFA}	0.0444 (0.0316 , 0.0571)	0.0803 (0.041 , 0.13)
	Time delay for production of G-3-P from glucose.	$\tau_{\text{G-3-P}}$	33.095 (-28.589 , 94.779)*	33.24 (-75.768 , 142.248)*
	Fraction of adipose glucose coverted	frac_{use}	0.919 (0.279 , 1.559)†	0.706 (0.048 , 1.364)†
