# Supplementary material for: A computational model of postprandial adipose tissue lipid metabolism derived using human arteriovenous stable isotope tracer data
Source: PLoS Comput Biol. 2019 Oct 3;15(10):e1007400. doi: 10.1371/journal.pcbi.1007400 (PMC6890259; doi:10.1371/journal.pcbi.1007400)
Supplement: S1 File — (PDF) [file pcbi.1007400.s007.pdf]

1 Supplementary Section S1: Sensitivity Analysis

Supporting information file S1 for:
A computational model of postprandial adipose tissue lipid metabolism derived using human arterio-venous stable isotope tracer data.

Shauna D. O'Donovan, Michael Lenz, Roel G. Vink, Nadia J.T. Roumans, Theo M.C.M de Kok, Edwin C.M Mariman, Ralf L.M. Peeters, Natal A.W. van Riel Marleen A. van Baak, Ilja C.W. Arts.

Sensitivity Analysis
In order to evaluate the sensitivity of the refined model to each parameter, having estimated an optimal parameter set, the value of each parameter was varied, in turn, through a range plus or minus 25% of the optimal value for that parameter. The results are shown below (Fig S5 - Fig S 18). For each sensitivity plot, red crosses indicate the mean calculated metabolite flux at a given time point, error bar show the standard error of the respective mean. Black lines represent the model prediction at baseline using the optimal parameter set. The colour of the model simulations generate in the sensitivity analysis correspond to the value of the varied parameter used for that simulation, this is indicated on the colour bar accompanying each figure.

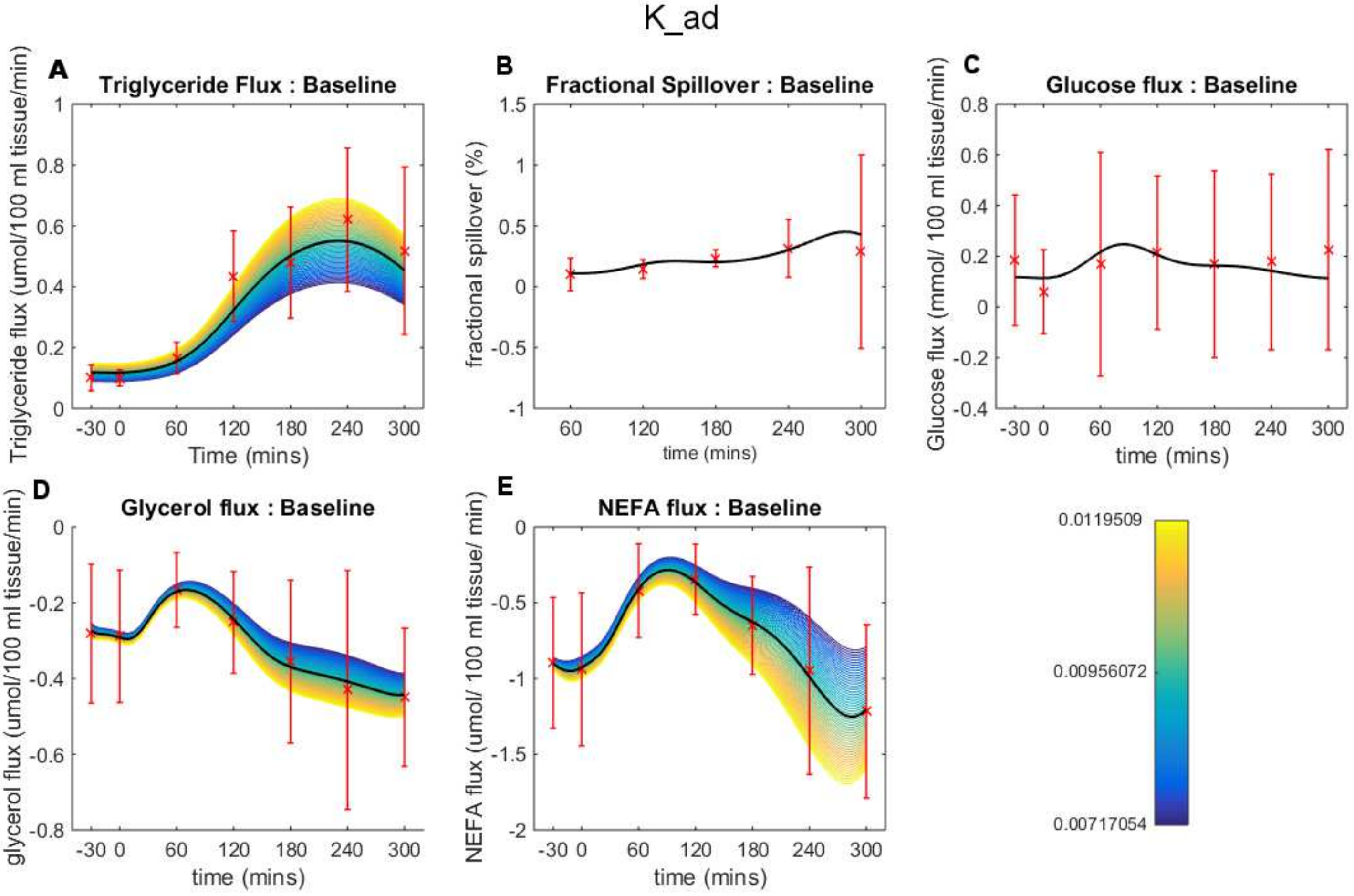

Supplementary Fig S4 : Sensitivity analysis for K_{ad}.
A 25% variation from the optimal value for the parameter K_{ad} has a strong effect on the model predictions for triglyceride, glycerol, and NEFA flux, the model terms in which it appears, with no effect on the fractional spill-over or glucose flux. As K_{ad} is the rate parameter for the LPL mediated lipolysis of circulating triglyceride, releasing NEFA and glycerol, the effect of varying this parameter on the triglyceride, glycerol, and NEFA flux is not surprising. Variation in the value of K_{ad} shifts the model predicted fluxes up or down in a uniform manner, having its strongest effect in the 120-300 minute interval when the concentrations of plasma triglyceride and LPL delayed insulin are reaching their respective peaks. It does not affect the time at which peak flux occur, only the magnitude of the peak, reflecting the linear structure of the LPL lipolysis model term.

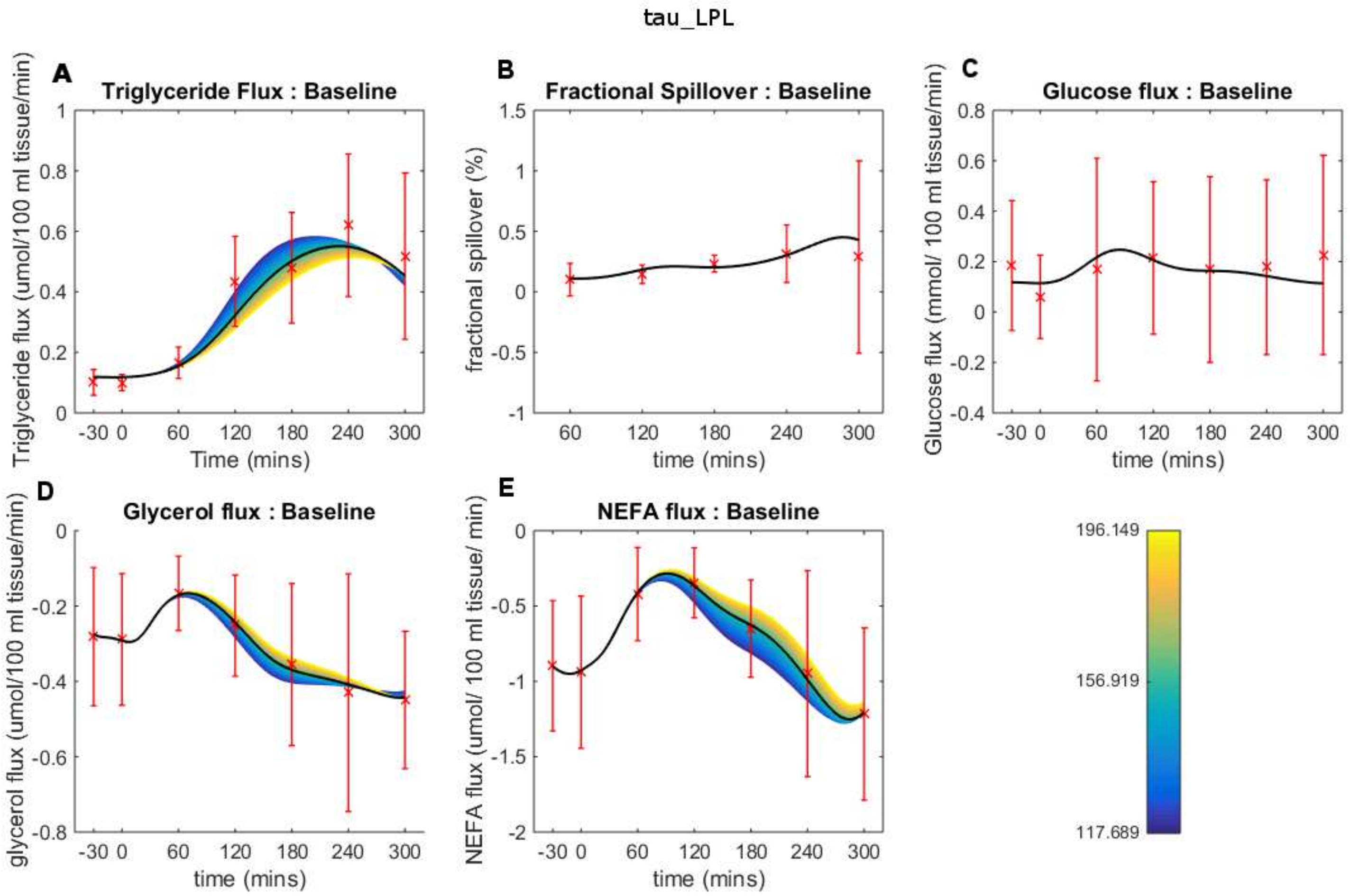

Supplementary Figure S5 : Sensitivity analysis for τ_{LPL}.
A 25% variation from the optimal value for the parameter τ_{LPL}. has a strong effect on the model predictions for triglyceride, glycerol, and NEFA flux, the terms in which it appears, with no effect on the fractional spill-over or glucose flux. τ_{LPL} is the time delay parameter for the insulin signal stimulating LPL lipolysis of circulating triglyceride releasing glycerol and NEFA, as with K_{ad}, it is not unexpected that variations in this parameter value would have an effect on the model predictions of the triglyceride, glycerol, and NEFA fluxes. Note, the pattern of the effect is very different to that of variation in K_{ad}, variation in the value of τ_{LPL}. effects both the magnitude and time of the peak in model predictions of the triglyceride flux as a result of the dampening of the insulin signal, with little to no effect on the fasting state. The effect on varying the insulin delay of LPL lipolysis on the hydrolysis of circulating triglyceride is then propagated into the glycerol and NEFA fluxes.

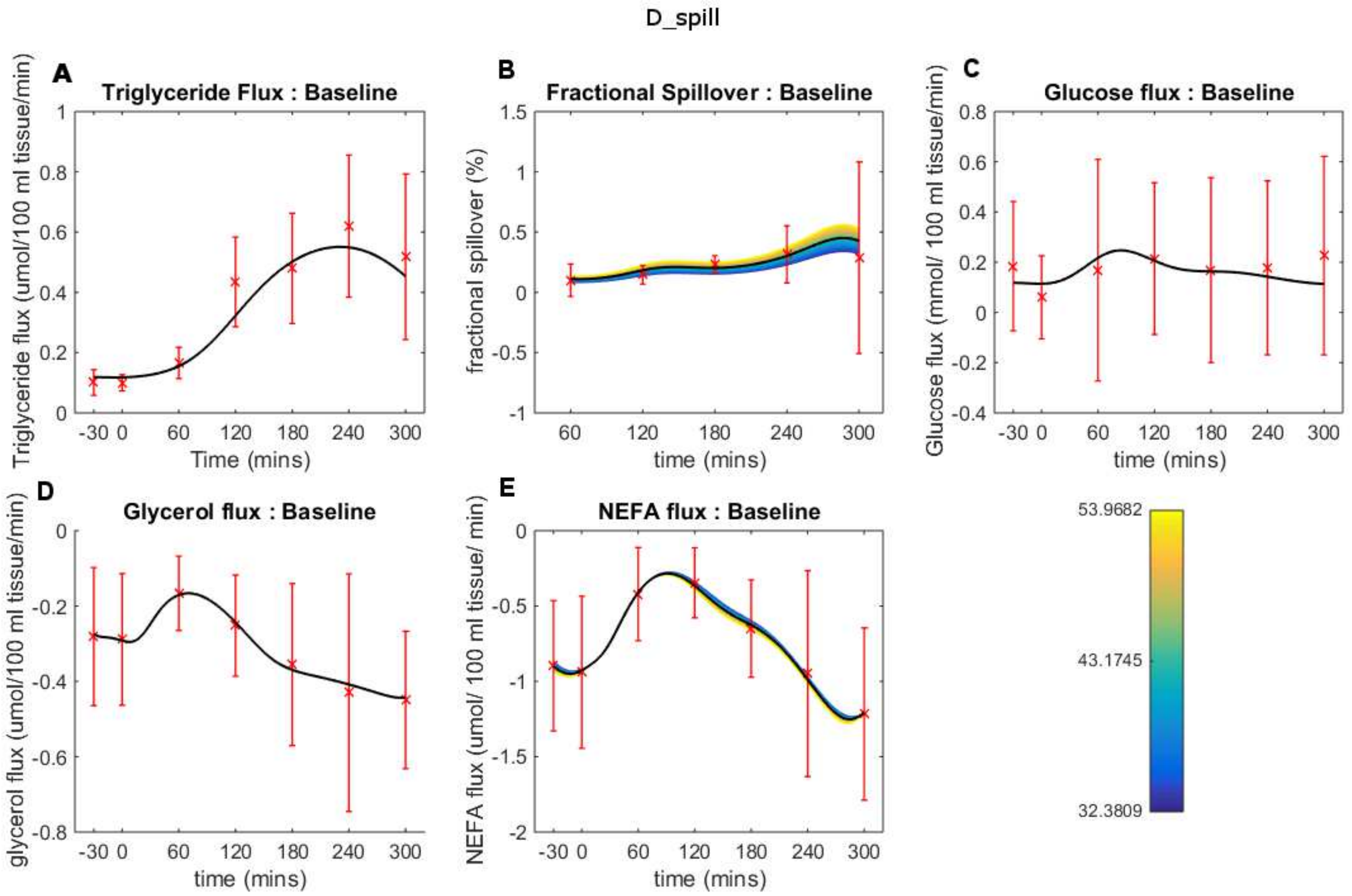

Supplementary Fig S6 : Sensitivity analysis for D_{spill}.
A 25% variation from the optimal value for the parameter D_{spill} has a moderate effect on the model predictions for the fractional spill-over with no effect on the triglyceride, glucose, and glycerol fluxes and a very small effect on the NEFA flux in the fasting and late post-prandial period. Increasing the value of D_{spill} from the optimal value produces a corresponding increase in the model prediction of fractional spill-over (a 25% increase in the value for D_{spill} produces approximately a 25% increase in the model prediction of fractional spill-over at 300 minutes following meal consumption) while decreasing the value of D_{spill} reduces it.

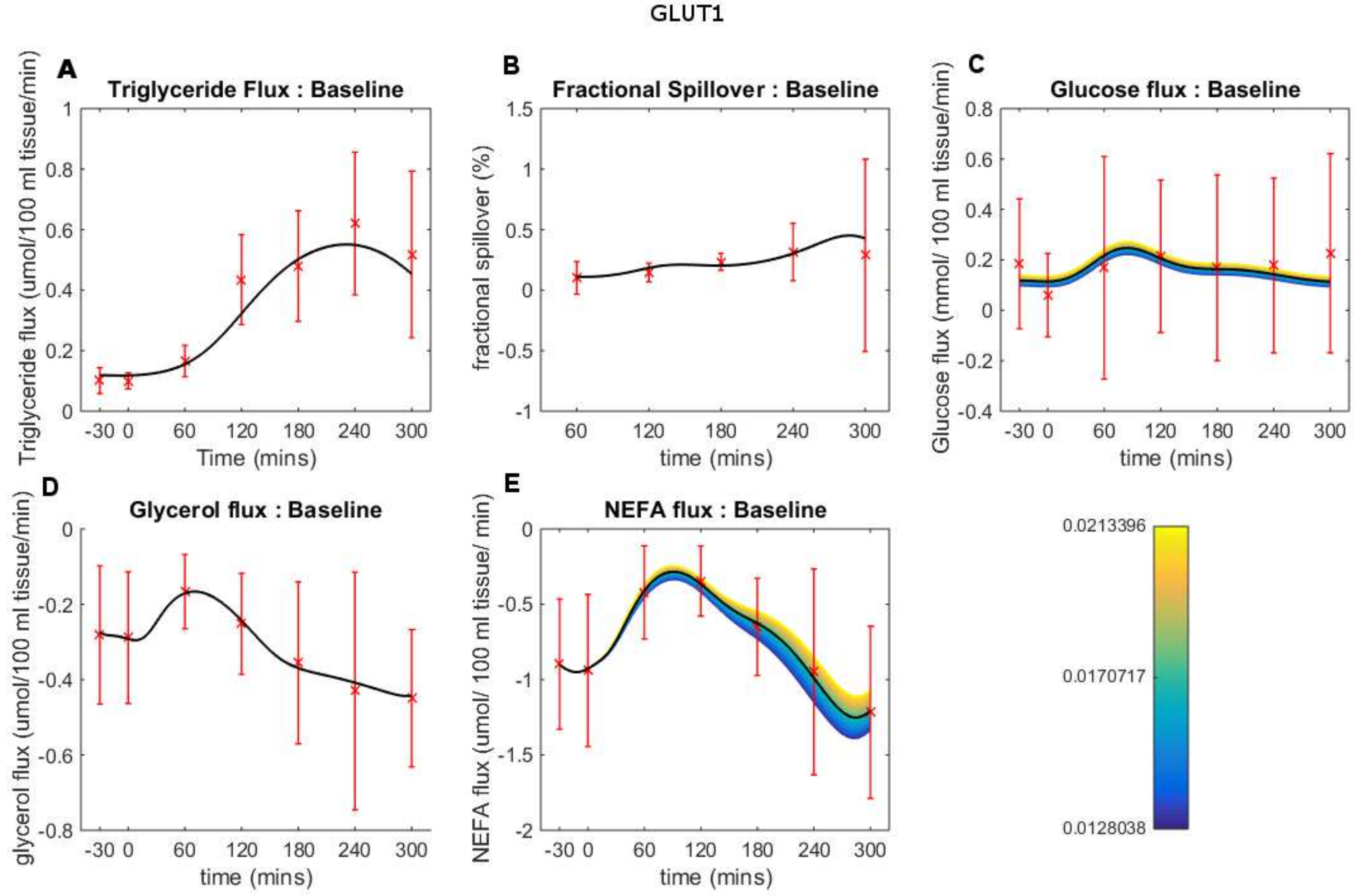

Supplementary Fig S7 : Sensitivity analysis for GLUT1.
A 25% variation from the estimated optimal value for the parameter GLUT1 has a modest effect on the model predictions for glucose flux, which is to be expected, a strong effect on the model prediction for the post-prandial NEFA flux and little to no effect on the fractional spill-over, triglyceride and NEFA fluxes. As GLUT1 is rate coefficient parameter for insulin independent glucose uptake by the adipose tissue the effect of varying the GLUT1 value on the model prediction of the glucose flux is uniform, it simply shifts the curve up or down. As, in this model, adipose tissue glucose supplies the G-3-P necessary for re-esterification, alterations in the glucose uptake by the adipose tissue due to variation in the value of GLUT1 result in large variations in the NEFA flux as the rate of re-esterification will be inhibited or increased. Changes in glucose flux appear to have little effect on re-esterification in the fasting period. Given the strong effect of variation of GLUT1 glucose uptake on the post-prandial NEFA flux, it would suggest availability of G-3-P becomes rate limiting on the rate of re-esterification as greater pressure is put on the system to remove excess lipids from circulation. In our refined model glucose is the sole source of the G-3-P used in re-esterification, glyceroneogenesis may also provide a source of G-3-P reducing the rate limiting effect of glucose uptake on the rate of re-esterification.

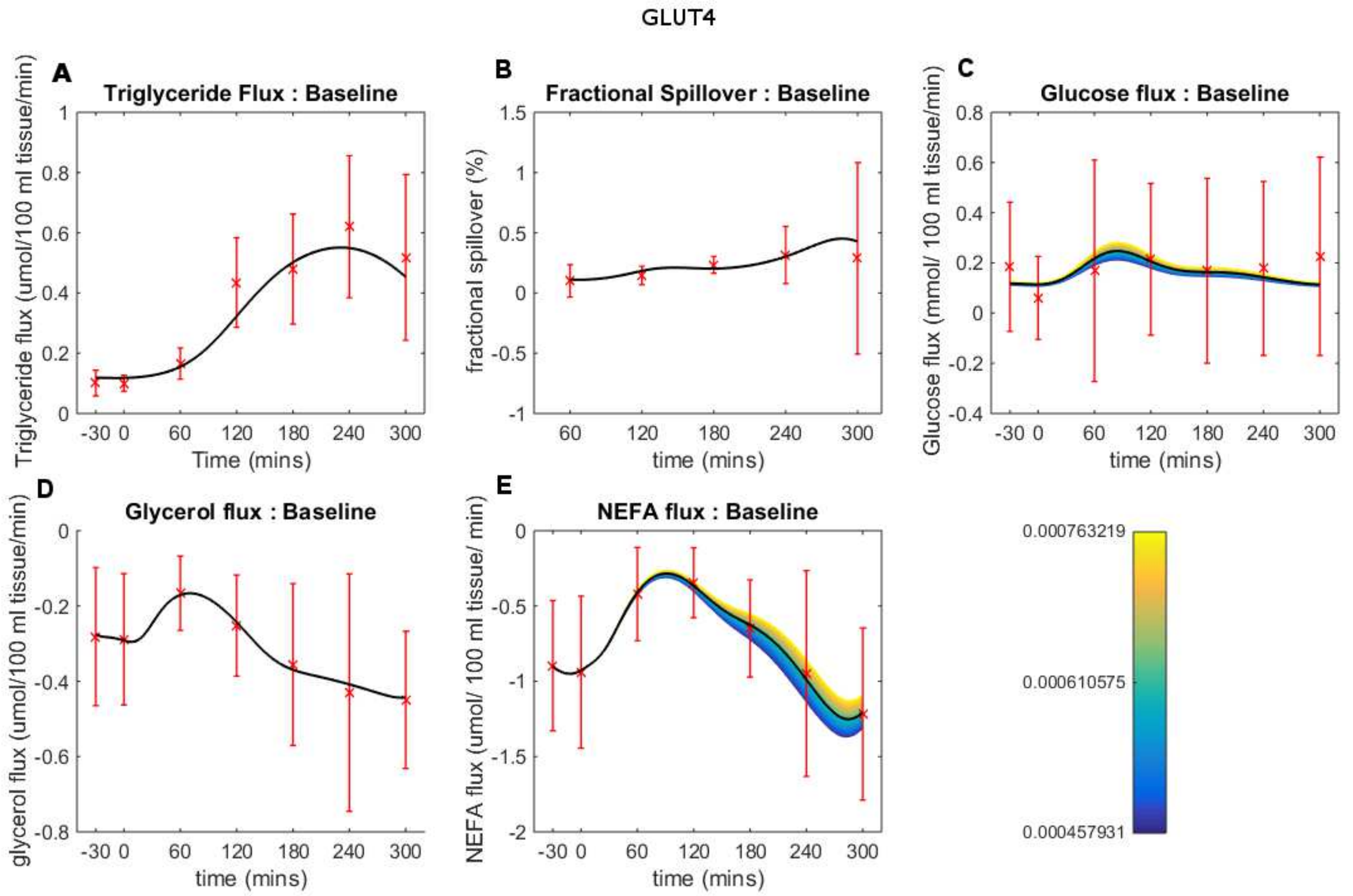

Supplementary Fig S8: Sensitivity analysis for GLUT4.
As with variation of GLUT1, 25% variation from the estimated optimal value for the parameter GLUT4 has a modest effect on the model predictions for glucose flux, a strong effect on the model prediction for the post-prandial NEFA flux and little to no effect on the fractional spill-over, triglyceride and NEFA fluxes. GLUT4 is rate coefficient for insulin dependent glucose uptake by the adipose tissue. As a result, variation in the value of GLUT4 has a more pronounced effect around 60 mins postprandially, when the delayed adipose tissue insulin signal would be at its peak. As with variation of GLUT1, variation of GLUT4 results in a large effect on the NEFA flux predictions of the refined model as adipose glucose supplies the source of G-3-P necessary for re-esterification.

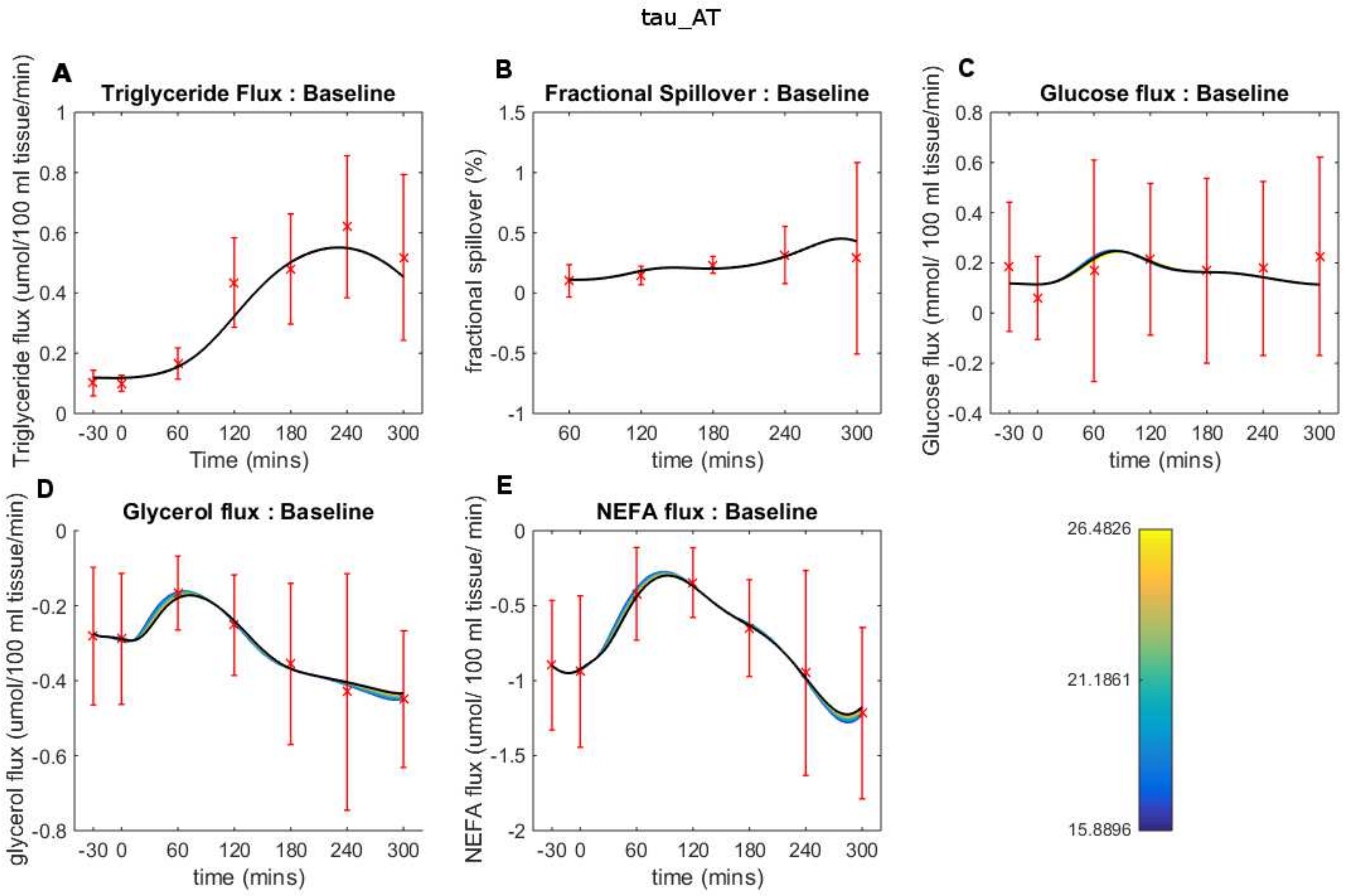

Supplementary Fig S9a : 25% sensitivity analysis for τ_{AT}.
A 25% variation from the estimated optimal value for the parameter τ_{AT} has minor effects on model prediction of the glycerol and NEFA fluxes. τ_{AT} is the time delay parameter for the adipose tissue insulin signal.

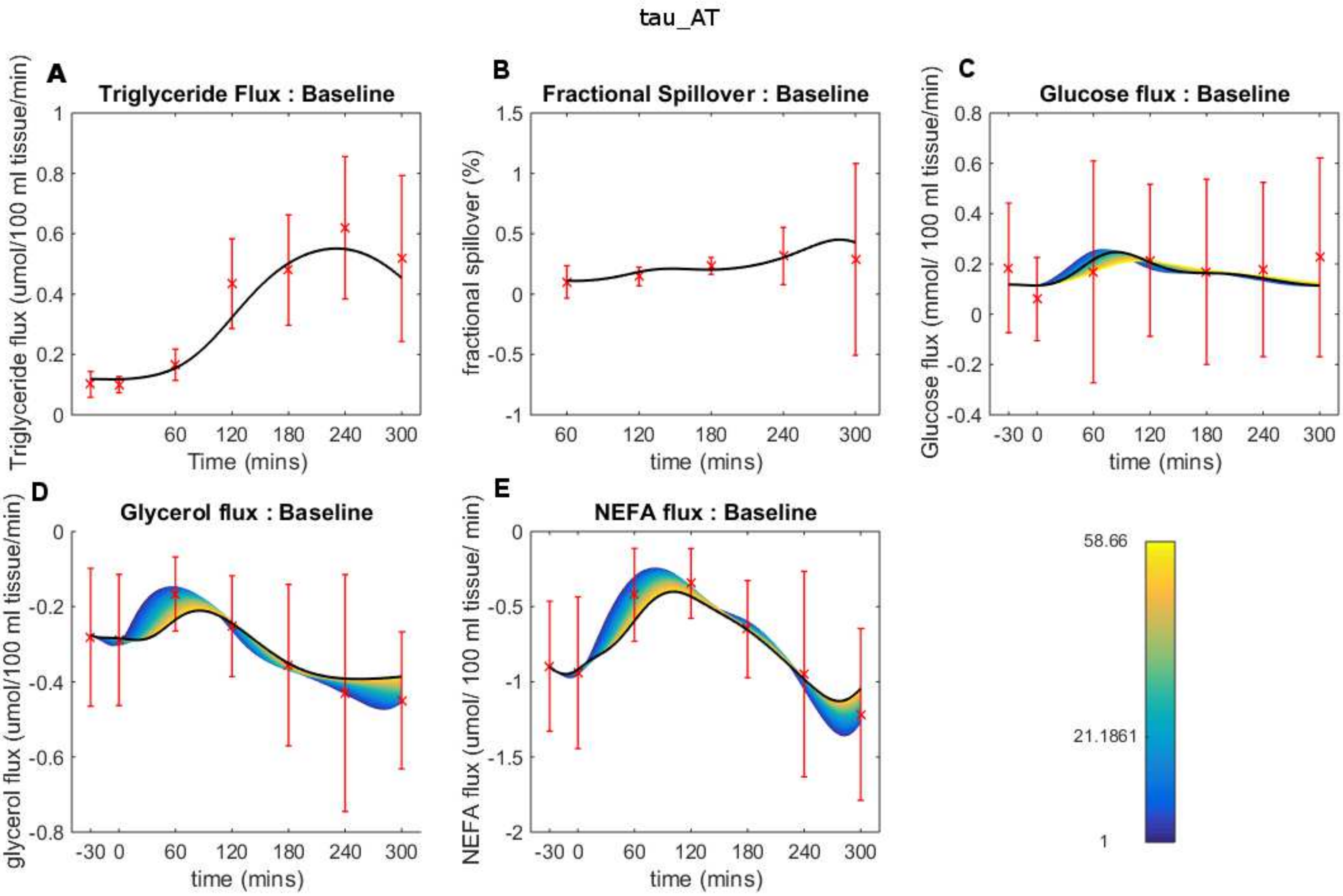

Supplementary Fig S9b : Sensitivity analysis for τ_{AT} through 95% confidence interval.
Repeating the sensitivity analysis, varying the value of τ_{AT} through its 95% confidence interval of 58.7 minutes. Here we see the stronger effect of further dampening the adipose tissue insulin signal on the glycerol and NEFA fluxes and begin to see an effect on the glucose flux as a result of delayed stimulation of the GLUT4 mediated uptake. Reduced values for τ_{AT} result in faster insulin signalling in the inhibition of ATL lipolysis and stimulation of re-esterification, which is evident with the swifter decrease in both the glycerol and NEFA efflux following consumption of the meal.

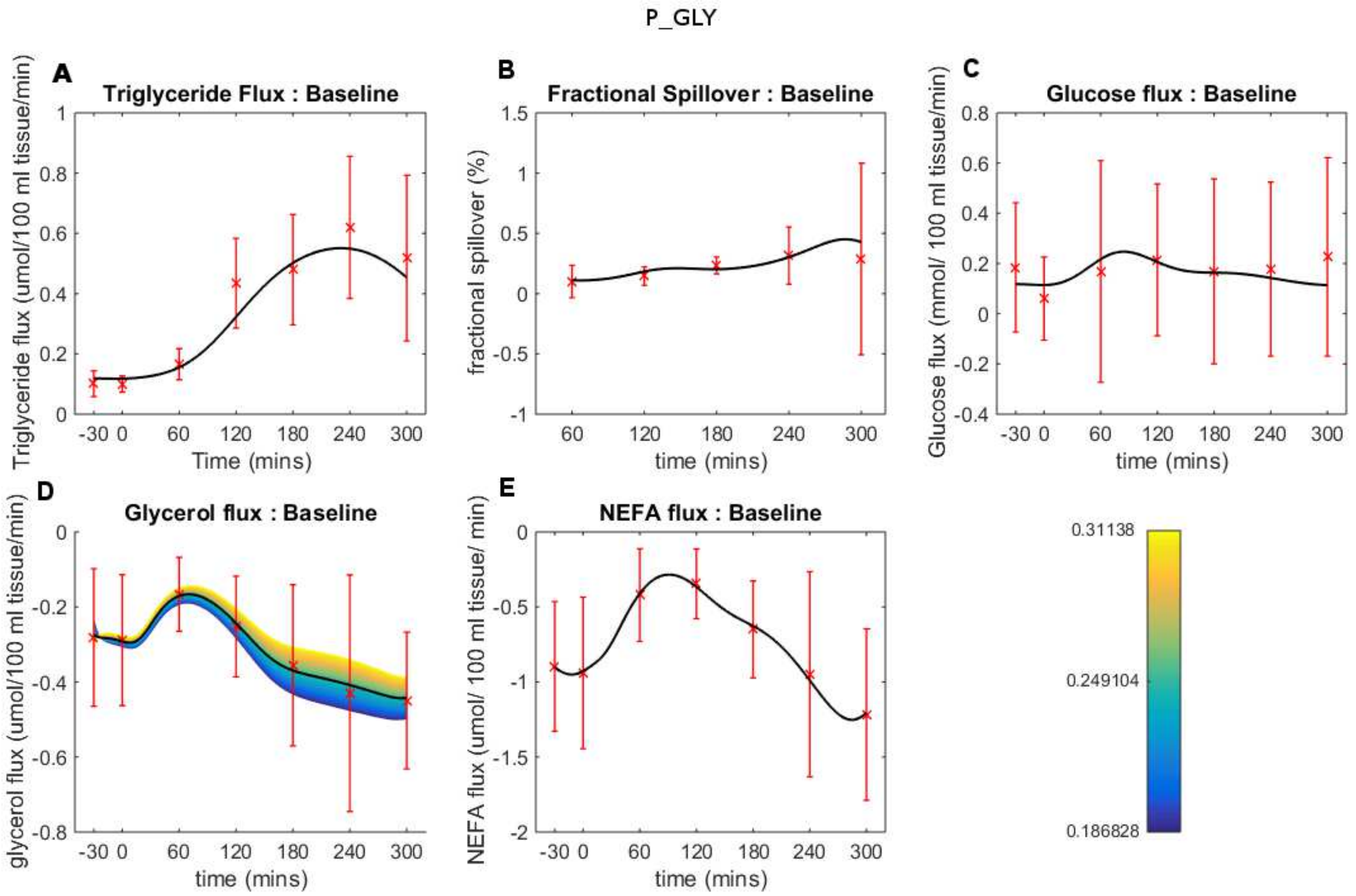

Supplementary Fig S10: Sensitivity analysis for P_{GLY}.
A 25% variation from the estimated optimal value for the parameter P_{GLY} has a strong effect on the glycerol flux but no effect on the triglyceride, glucose, and NEFA fluxes, nor the fractional spill-over. P_{GLY} is the rate parameter for the concentration gradient dependent uptake and release of glycerol by the adipose space from the plasma. As glycerol is a by-product of LPL and ATL lipolysis it is not rate limiting in any of these reactions. Variations in P_{GLY} have the strongest effect in the later post-prandial period, when plasma concentrations of glycerol are much higher than adipose tissue concentration due to increased LPL lipolysis, it is at this point that the influx of glycerol to the adipose space is at its highest.

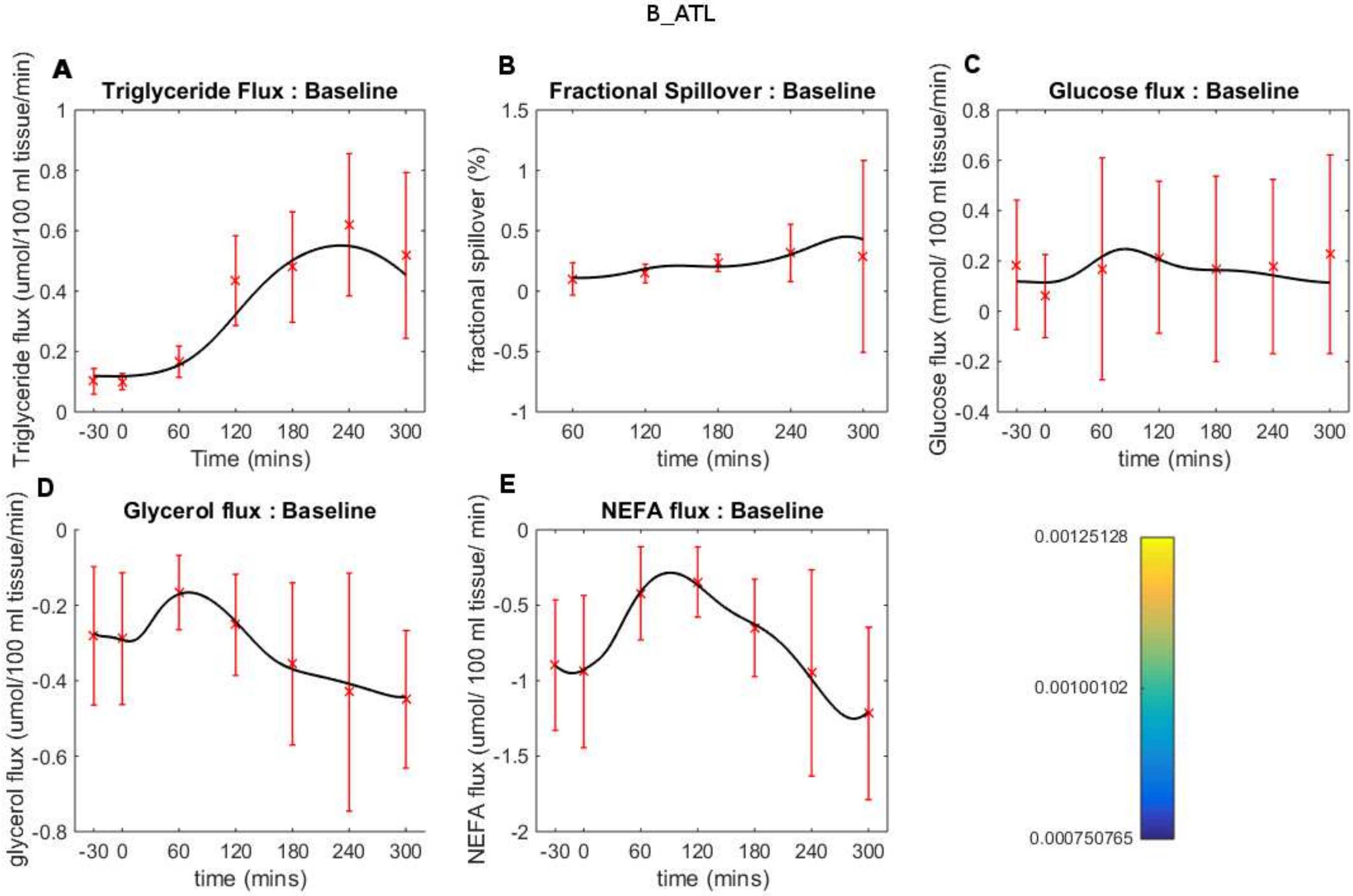

Supplementary Fig S11a: 25% Sensitivity analysis for B_{ATL}.
A 25% variation from the estimated optimal value for the parameter B_{ATL} has little to no effect on any of the model predictions for measured fluxes within the Yoyo study. Very slight variation can be seen in the glycerol and NEFA fluxes, which may be expected as B_{ATL} is the rate parameter for basal ATL lipolysis within the adipose space releasing NEFA and glycerol. As the estimated value of B_{ATL} is quite small, 0.001 mmol/ml/min, a 25% variation in the parameter value is not that large.

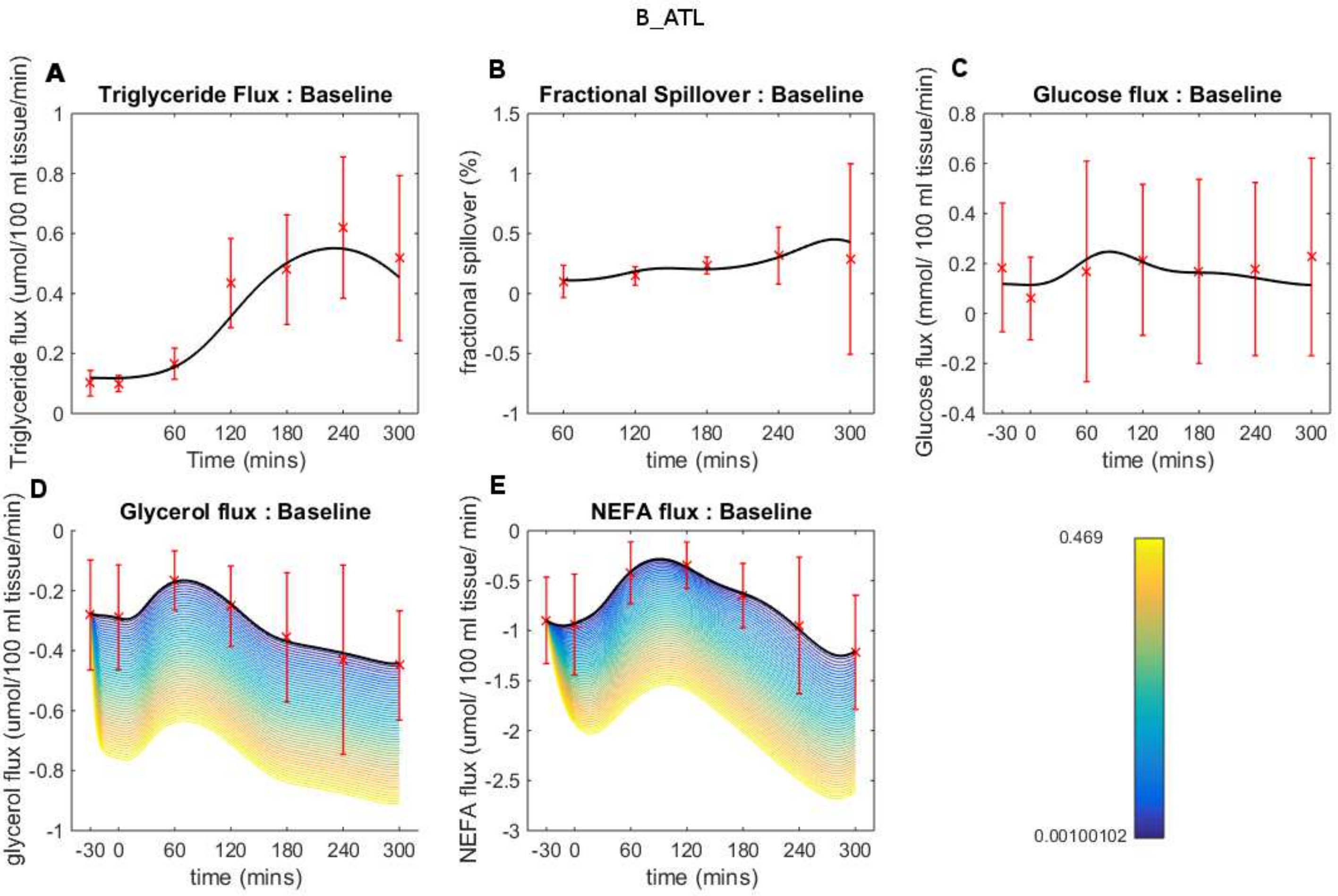

Supplementary Fig S11b: Sensitivity analysis for B_{ATL} through 95% confidence interval.
Varying B_{ATL} through its 95% confidence interval we see a more pronounced linear effect on the glycerol and NEFA fluxes.

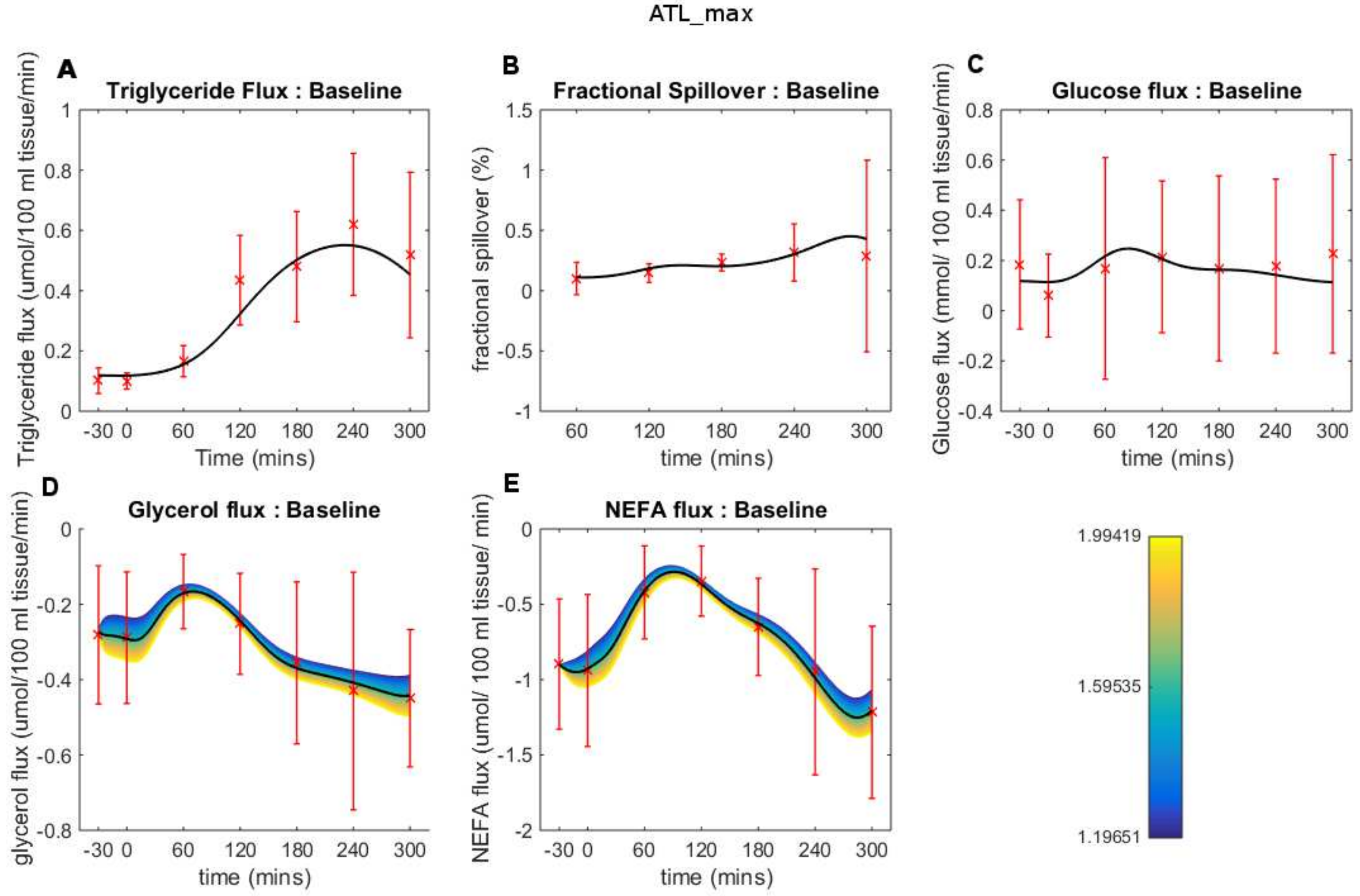

Supplementary Fig S12 : Sensitivity analysis for ATL_{max}.
A 25% variation from the estimated optimal value for the parameter ATL_{max} has noticeable effect on the glycerol and NEFA fluxes while having no effect on the triglyceride and glucose flux nor the fractional spill-over. ATL_{max} is the maximum rate of ATL lipolysis of triglyceride stored within the adipose tissue which has been described in this model using Michaelis-Menten kinetics. Variation in the value for ATL_{max} has its greatest impact on the glycerol and NEFA flux in the fasting and later postprandial period, when the adipose tissue insulin signal is at its maximum. As ATL lipolysis is inhibited by the delayed adipose tissue insulin signal the rate of lipolysis is lower in the early postprandial period. As a result increases in ATL_{max} have little effect, large reductions (25%) in the value of ATL_{max} have minor effects. In the fasting and later postprandial periods the adipose tissue insulin signal is lower, thus the rate at which ATL lipolysis can occur is more sensitive to changes in the value for ATL_{max}.

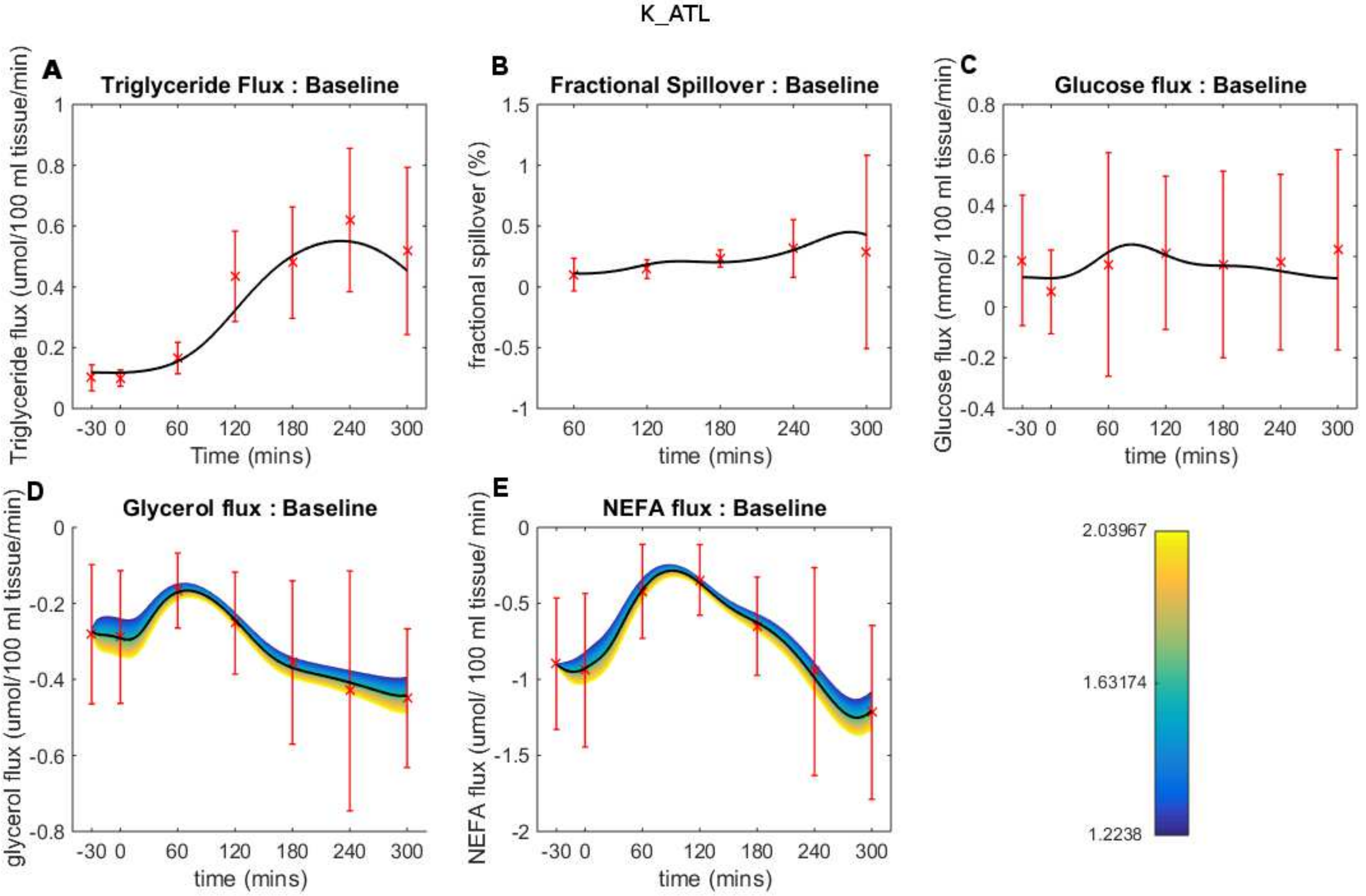

Supplementary Fig S13 : Sensitivity analysis for K_{ATL}.
A 25% variation from the estimated optimal value for the parameter K_{ATL} has noticeable effect on the glycerol and NEFA fluxes while having no effect on the triglyceride and glucose flux nor the fractional spill-over. K_{ATL} is the Michaelis-Menten coefficient of ATL lipolysis of triglyceride stored within the adipose tissue. K_{ATL} governs the rate at which saturation is reached. As with ATL_{max} changes in K_{ATL} have a stronger effect on model predictions of the glycerol and NEFA fluxes in the fasting and late postprandial period when the effect of adipose tissue insulin inhibition is less and the reaction is expected to reach saturation.

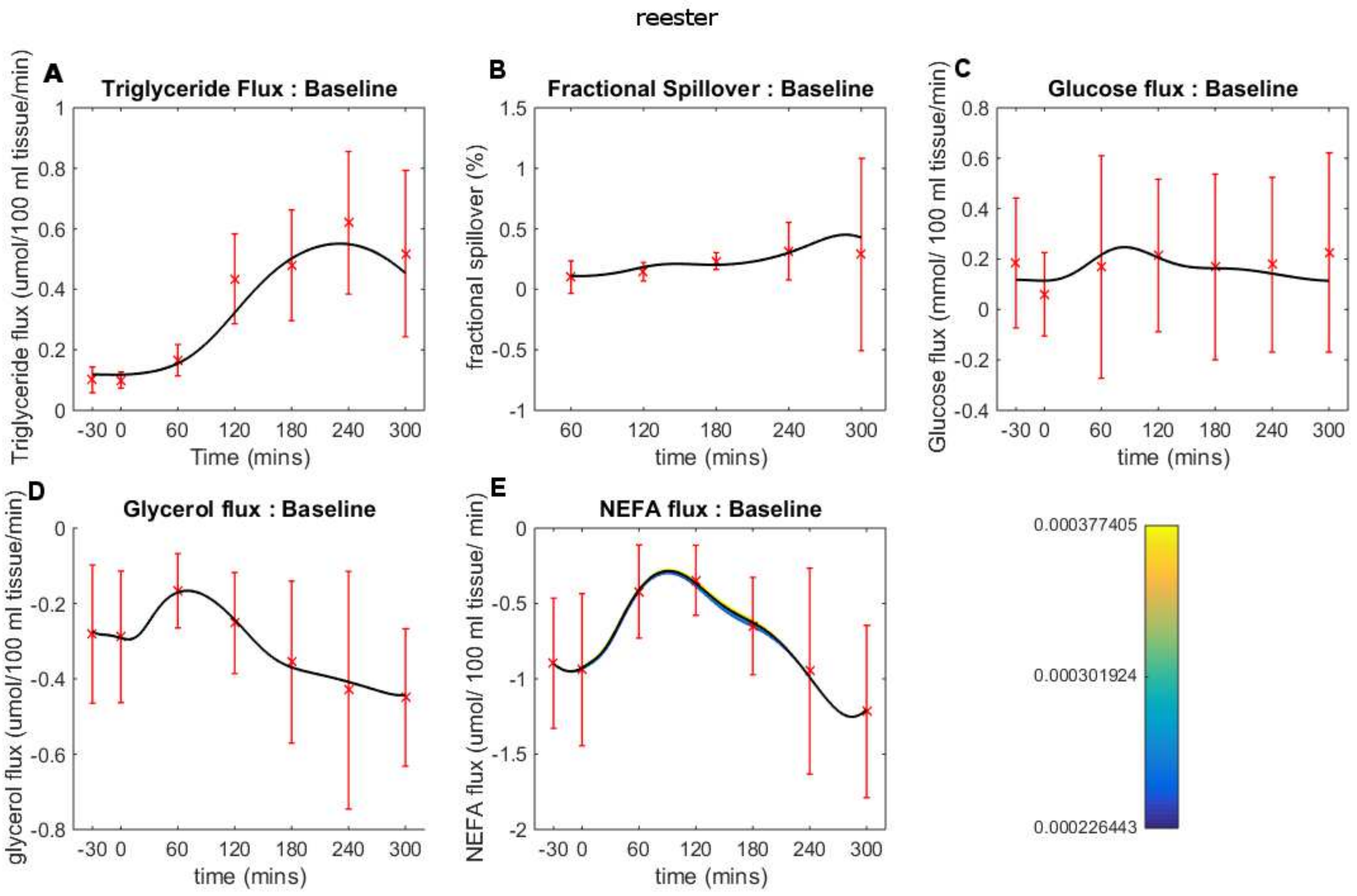

Supplementary Fig S14 : Sensitivity analysis for $K_{reester}$.
A 25% variation in the value for the parameter reester from its optimal value has no effect on any of the fluxes or fractional spill-over predicted by the model. $K_{reester}$ is the rate parameter governing the rate of re-esterification within the adipose space, a reaction dependent on the adipose space concentration of G-3-P and NEFA and is stimulated by the adipose insulin signal. As a portion of the glucose taken into the adipose space is use for production of G-3-P, determined by the parameter $frac_{use}$, the entire adipose G-3-P concentration is utilised in re-esterification. Thus, the rate of change of the adipose tissue concentration of G-3-P, equation 10, is equal to zero. Therefore, the rate of re-esterification, the product of $K_{reester}$ and adipose tissue concentration of NEFA and G-3-P, is equal to the rate of G-3-P production. Increases in the value of the parameter $K_{reester}$ are compensated for by a reduction in the model prediction of the adipose concentration of G-3-P resulting in no change in the rate of re-esterification.

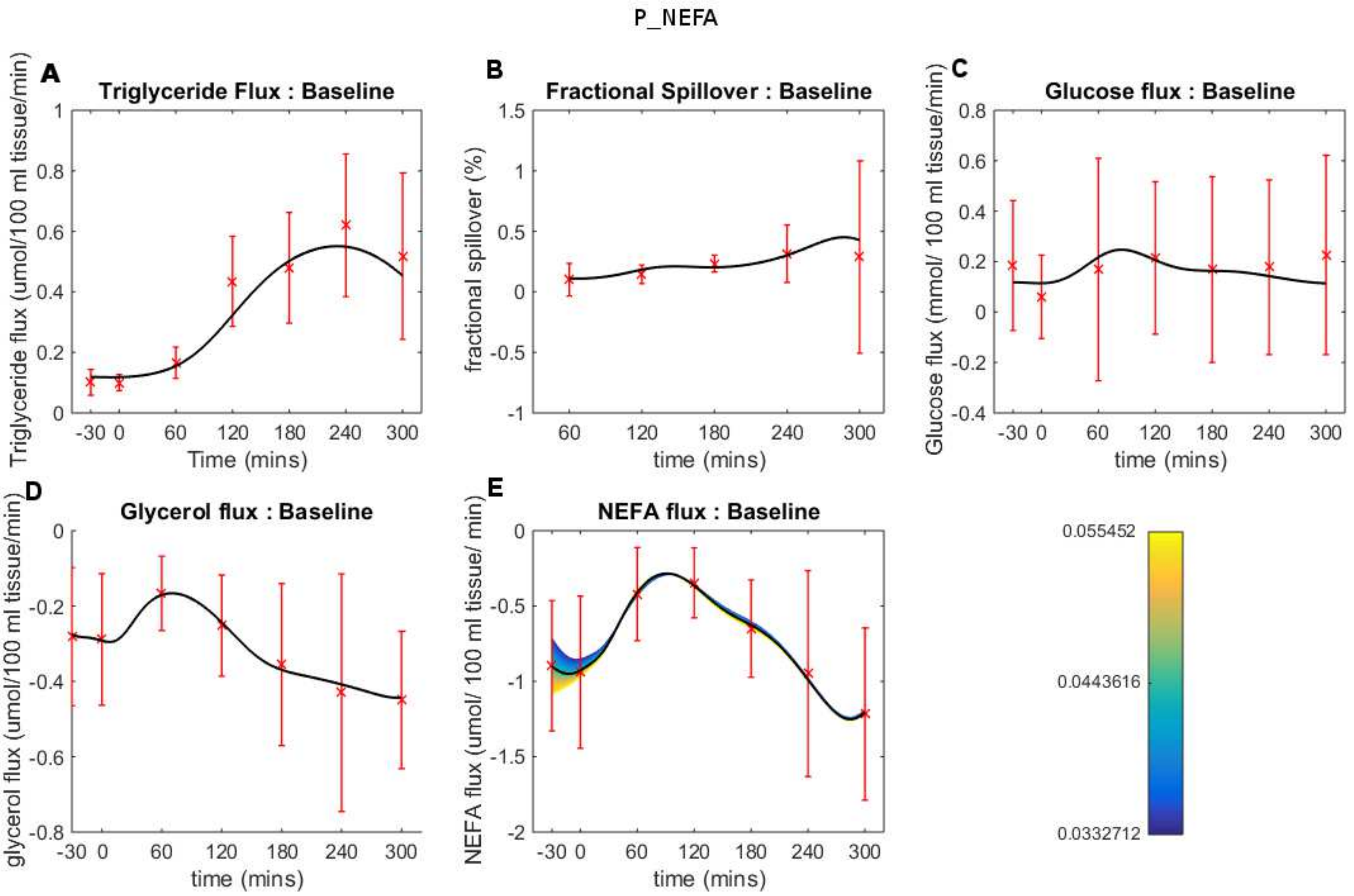

Supplementary Fig S15 : Sensitivity analysis for P_{NEFA}.
A 25% variation from the estimated optimal value for the parameter P_{NEFA} has an effect on the model prediction of the NEFA flux, which is to be expected and no effect on the remaining fluxes nor fractional spill-over. P_{NEFA} is the rate parameter governing the concentration gradient based uptake and release of NEFA by the adipose space. Variations in the value for P_{NEFA} have the strongest effect on the model prediction of the NEFA flux in the fasting period.

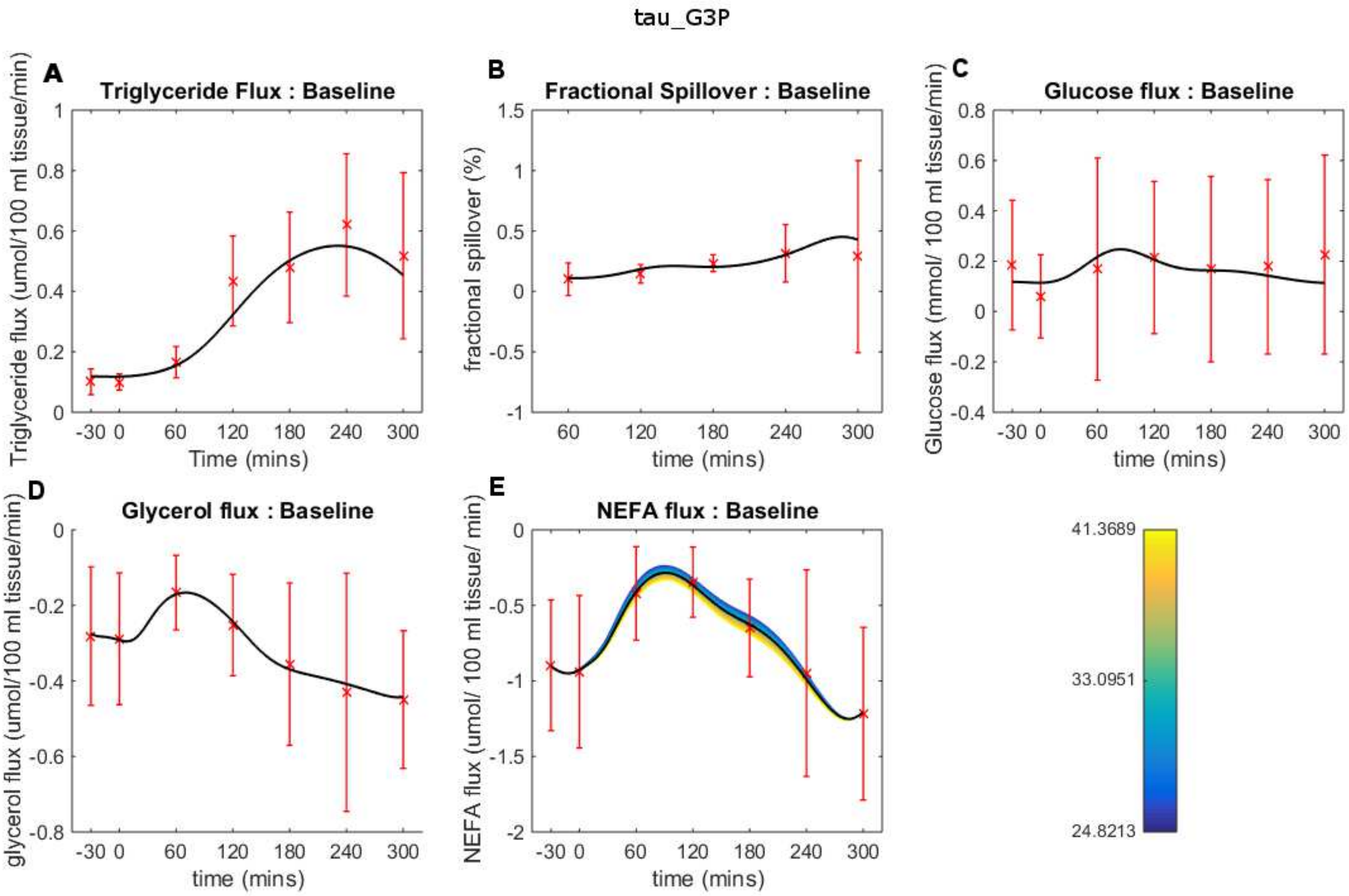

Supplementary Fig S16 : Sensitivity analysis for $\tau_{\text{G-3-P}}$.

A 25% variation from the optimal value for the parameter $\tau_{\text{G-3-P}}$ has a large effect on the model predictions for the early postprandial NEFA flux but no effect on the remaining model flux or fractional spill-over predictions. $\tau_{\text{G-3-P}}$ is the time delay parameter accounting for set of reactions necessary to convert a portion of the glucose taken up into the adipose space to G-3-P for use in re-esterification. Therefore, it is not surprising that variations in this time delay parameter will have an effect on the model predictions of the postprandial NEFA flux, as changes in the time necessary to convert the glucose taken up into G-3-P will impact on the maximum possible rate of re-esterification as it is a linear term.

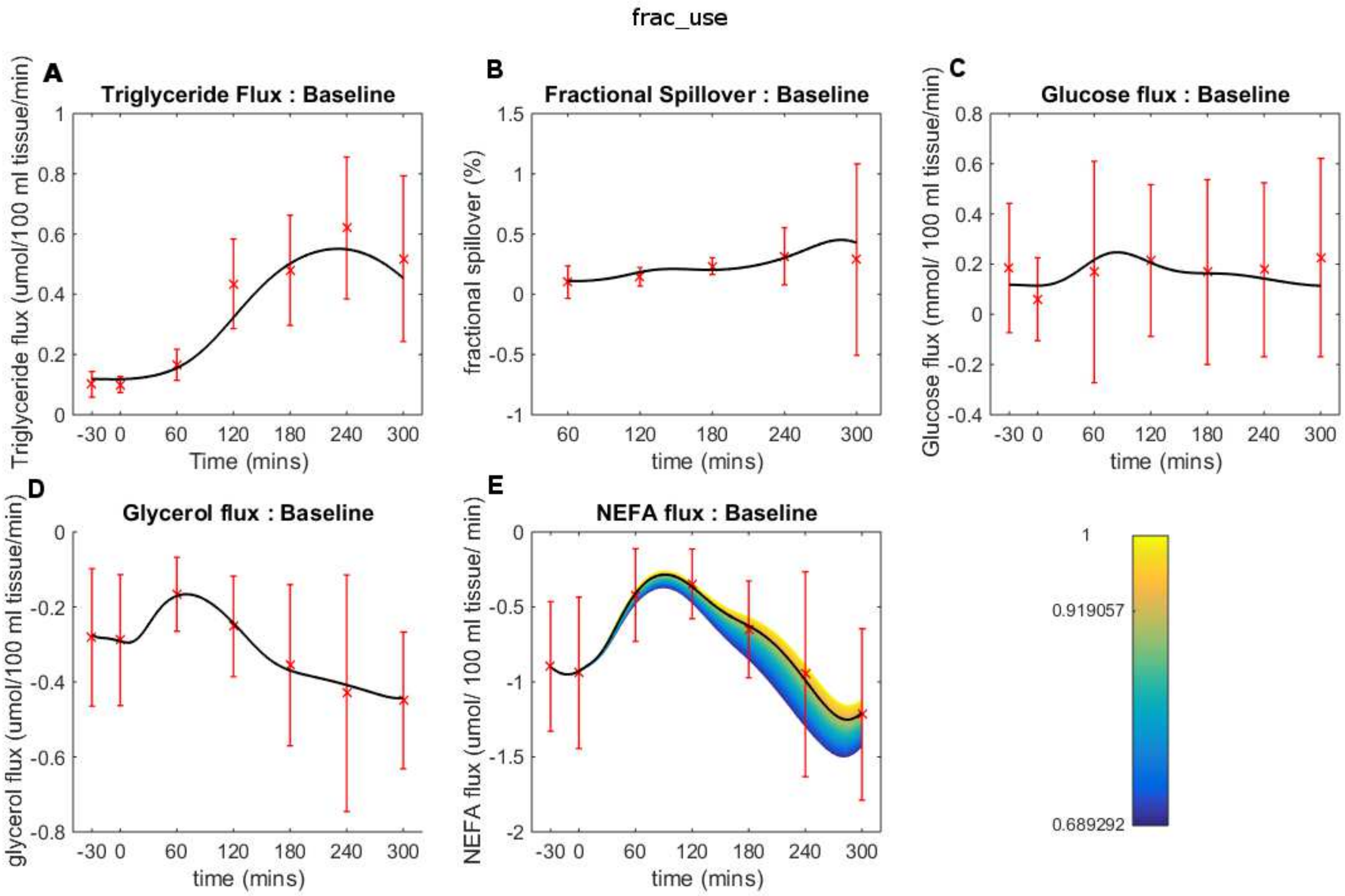

Supplementary Fig S17 : Sensitivity analysis $frac_{use}$**.**
A 25% variation from the optimal value for the parameter $frac_{use}$ has a strong effect on the model predictions for the glycerol flux and no effect on the remaining fluxes, nor the fractional spill-over predictions. The parameter $frac_{use}$ governs the portion of glucose taken into the adipose space which is converted to G-3-P for use in re-esterification, the remaining portion leaves the system for use in other processes within the adipose space. For $frac_{use}$ the value is reduced through 25% of the optimal value of 0.919. As $frac_{use}$ is a percentage it is bounded above by one and, hence, here in the sensitivity analysis is capped at one. Reducing the amount of glucose available for conversion to G-3-P and use in re-esterification reduces the rate of re-esterification, thereby increasing the NEFA efflux from the adipose space in the mid to late postprandial period. This further exemplifies the rate limiting effect of a source of G-3-P availability on re-esterification.
