# Supplementary material for: A computational model of postprandial adipose tissue lipid metabolism derived using human arteriovenous stable isotope tracer data
Source: PLoS Comput Biol. 2019 Oct 3;15(10):e1007400. doi: 10.1371/journal.pcbi.1007400 (PMC6890259; doi:10.1371/journal.pcbi.1007400)
Supplement: S2 File — (PDF) [file pcbi.1007400.s008.pdf]

1 Section S2 : Model Equations

Supporting information file S2 for:
A computational model of postprandial adipose tissue lipid metabolism derived using human arterio-venous stable isotope tracer data.

Shauna D. O'Donovan, Michael Lenz, Roel G. Vink, Nadia J.T. Roumans, Theo M.C.M de Kok, Edwin C.M Mariman, Ralf L.M. Peeters, Natal A.W. van Riel, Marleen A. van Baak, Ilja C.W. Arts.

Triglyceride flux

$$TG_{flux} = -K_{ad}[TG_{art}][I_{LPL}] \tag{1}$$

Fractional spill-over

$$\%\text{ spill-over} = \frac{1}{100}(D_{spill}\frac{I_B}{[I_{art}]}) \tag{2}$$

Glucose flux

$$G_{flux} = -GLUT1[G_{art}] - GLUT4[G_{art}][I_{AT}] \tag{3}$$

Production of adipose G-3-P

$$\frac{d[\text{G-6-P}]}{dt} = \frac{1}{\tau_{\text{G-3-P}}}(2frac_{use}(GLUT1[G_{art}] + GLUT4[G_{art}][I_{AT}]) - [\text{G-6-P}]) \tag{4}$$

$$\frac{d[\text{G-3-P}_{pro}]}{dt} = \frac{1}{\tau_{\text{G-3-P}}}([\text{G-6-P}] - [\text{G-3-P}_{pro}]) \tag{5}$$

$$\frac{d[\text{G-3-P}_{AT}]}{dt} = [\text{G-3-P}_{pro}] - K_{reest}[I_{AT}][NEFA_{AT}][\text{G-3-P}_{AT}] \tag{6}$$

Glycerol flux

$$GLY_{flux} = K_{ad}[TG_{art}][I_{LPL}] + p_{GLY}([GLY_{AT}] - ([GLY_{art}] + K_{ad}[TG_{art}][I_{LPL}])) \tag{7}$$

$$\frac{d[GLY_{AT}]}{dt} = -p_{GLY}([GLY_{AT}] - [GLY_{art}] + K_{ad}[TG_{art}][I_{LPL}]) + B_{ATL} + \frac{ATL_{max}}{1 + \frac{[I_{AT}]}{K_{ATL}}} \tag{8}$$

NEFA flux

$$\begin{aligned} NEFA_{flux} = {} & \frac{3}{100}(D_{spill}\frac{I_B}{[I_{art}]})K_{ad}[TG_{art}][I_{LPL}] \\ & - p_{NEFA}(([NEFA_{art}] + \frac{3}{100}(D_{spill}\frac{I_B}{[I_{art}]})K_{ad}[TG_{art}][I_{LPL}]) - [NEFA_{AT}]) \end{aligned} \tag{9}$$

$$\begin{aligned} \frac{d[NEFA_{AT}]}{dt} = {} & \frac{3}{100}(1 - D_{spill}\frac{I_B}{[I_{art}]})K_{ad}[TG_{art}][I_{LPL}] \\ & + p_{NEFA}(([NEFA_{art}] + \frac{3}{100}(D_{spill}\frac{I_B}{[I_{art}]})K_{ad}[TG_{art}][I_{LPL}]) - [NEFA_{AT}]) \\ & + 3(B_{ATL} + \frac{ATL_{max}}{1 + \frac{[I_{AT}]}{K_{ATL}}}) - 3(K_{reester}[I_{AT}][NEFA_{AT}][\text{G-3-P}_{AT}]) \end{aligned} \tag{10}$$

LPL insulin signal

$$\frac{d[I_1]}{dt} = \frac{1}{\tau_{LPL}}([I_{art}] - [I_1]) \tag{11}$$

$$\frac{d[I_2]}{dt} = \frac{1}{\tau_{LPL}}([I_1] - [I_2]) \tag{12}$$

$$\frac{d[I_{LPL}]}{dt} = \frac{1}{\tau_{LPL}}([I_2] - [I_{LPL}]) \tag{13}$$

Adipose tissue insulin signal

$$\frac{d[I_1]}{dt} = \frac{1}{\tau_{AT}}([I_{art}] - [I_1]) \tag{14}$$

$$\frac{d[I_2]}{dt} = \frac{1}{\tau_{AT}}([I_1] - [I_2]) \tag{15}$$

$$\frac{d[I_{AT}]}{dt} = \frac{1}{\tau_{AT}}([I_2] - [I_{AT}]) \tag{16}$$
