# Supplementary material for: A computational model of postprandial adipose tissue lipid metabolism derived using human arteriovenous stable isotope tracer data
Source: PLoS Comput Biol. 2019 Oct 3;15(10):e1007400. doi: 10.1371/journal.pcbi.1007400 (PMC6890259; doi:10.1371/journal.pcbi.1007400)
Supplement: S3 File — (ZIP) [file pcbi.1007400.s009.zip › Computational_model_adipose_metabolism/READ ME.pdf]

The code for the implementation of the adipose tissue model described in this article is provided as Matlab m-files (MATLAB 2014b, The MathWorks Inc., Natick, Massachusetts, United States.) The provided folder contains implementations of the adipose tissue model, parameter estimation, model analysis and visualisation. The folder also contains a sample data file *"sample_data.mat"*. This file contains a Matlab structure consisting of time series of simulated postprandial adipose tissue flux data and arterial metabolite concentrations in the format required for the implementation of the model. The scripts for parameters estimation and flux predictions are outlined below.

Estimating_Model_Parameters_Script.m

This script facilitates the estimation of model parameters from adipose tissue flux data. Allows the user to specify input data containing both the dependent inputs for the model simulation (arterial metabolite concentrations) and adipose tissue flux data to which they wish to fit the model (default is set to upload the sample data file). The script calls up the function *"Parameter_Fit_Adipose_Model.m"* which estimates the parameter values using *lsqnonlin* a local, gradient based, least-squares solver. Within the *Estimating_Model_Parameters_Script* the user can specify upper and lower bounds for the parameter search and an initial set of parameter values for the *lsqnonlin* algorithm. Options for the *lsqnonlin* least-squares solver (algorithm = 'TrustReflectiveRegion', and stopping tolerances MaxFunEvals = 500 and TolX=1e-25) are defined within the *Parameter_Fit_Adipose_Model* function. There is an option to generate a figure of the model fit to the data by setting the value of the variable fig_yn = 1.

Flux_Predictions_Script.m

This script allows for the prediction of adipose tissue fluxes using the model for user supplied parameter values and arterial metabolite concentrations. The user can upload data in the form of an input data structure or manually define each element in the input data structure. Individual parameter values can be specified, by default these are set to the optimal value for the provided sample data, or a vector of parameter value be specified. Initial values for adipose tissue concentrations of glycerol, G-3-P, and NEFA also need to be specified. The time span for simulation of adipose tissue fluxes must also be set. There is an option to generate a figure of the model prediction of adipose tissue flux given the parameter set and arterial metabolite concentrations by setting the value of the variable fig_yn= 1.

Input structure

input_data.	TG_art	vector of time series of arterial triglyceride concentration.
	G_art	vector of time series of arterial glucose concentration.
	GLY_art	vector of time series of arterial glycerol concentration.
	NEFA_art	vector of time series of arterial NEFA concentration.
	I	vector of time series of arterial insulin concentration.
	t	vector of time points of sampling.
	labeling	label used in plotting results.
		additional data for parameter estimation.
	mean_TG_flux	vector of time series of mean adipose tissue triglyceride flux.
	std_TG_flux	vector of time series of the standard deviation in adipose tissue triglyceride flux.
	mean_spill	vector of time series of mean fractional spill-over of NEFA from LPL lipolysis of circulating triglyceride.
	std_spill	vector of time series of the standard deviation in fractional spill-over of NEFA from LPL lipolysis of circulating triglyceride.
	mean_G_flux	vector of time series of mean adipose tissue glucose flux.
	std_G_flux	vector of time series of the standard deviation in adipose tissue glucose flux.
	mean_GLY_flux	vector of time series of mean adipose tissue glycerol flux.
	std_GLY_flux	vector of time series of the standard deviation in adipose tissue glycerol flux.
	mean_NEFA_flux	vector of time series of mean adipose tissue NEFA flux.
	std_NEFA_flux	vector of time series of the standard deviation in adipose tissue NEFA flux.
